# Ultrasound-assisted Cu(II) Strecker-functionalized organocatalyst for green azide–alkyne cycloaddition and Ullmann reactions

**DOI:** 10.1038/s41598-024-62826-1

**Published:** 2024-05-27

**Authors:** Mahyar Aghajani, Minoo Dabiri

**Affiliations:** Department of Organic Chemistry and Oil, Faculty of Chemistry and Petroleum Sciences, Shahid Beheshti University, Tehran, 1983969411 Islamic Republic of Iran

**Keywords:** Strecker-functionalized surface, Nanocatalyst, Azide–alkyne cycloaddition, Ultrasonic promotion, 1,4-disubstituted 1,2,3-triazoles, Classic Ullmann reaction, Catalyst synthesis, Heterogeneous catalysis, Organocatalysis, Catalysis, Organic chemistry, Synthetic chemistry methodology, Chemistry, Chemical synthesis, Catalyst synthesis, Synthetic chemistry methodology, Nanoparticle synthesis

## Abstract

A new aminonitrile-functionalized Fe_3_O_4_ has been synthesized via the Strecker reaction, the designed aminonitrile ligand on the surface of the magnetic core coordinated to copper(II) to obtain the final new catalyst. The fabricated nanocatalyst was characterized by Fourier transform Infrared (FT-IR), Field Emission Scanning Electron Microscopy (FESEM), Energy-Dispersive X-ray spectroscopy (EDX), Transmission Electron Microscopy (TEM), Vibrating-Sample Magnetometer (VSM), Inductively Coupled Plasma Optical Emission Spectroscopy (ICP-OES), and Thermogravimetric Analysis (TGA). The high tendency of nitrogens in the aminonitrile functional group to make a complex with Cu(II) has caused the practical activity of this nucleus in this catalyst. This nanocatalyst performance was investigated in azide–alkyne Huisgen cycloaddition (3 + 2) reaction for achieving to 1,4-disubstituted 1,2,3-triazoles in water as a green media at room temperature. In another try, Classic Ullmann Reaction was investigated for the synthesis of biaryls at 85 °C promoted by ultrasonic condition (37 kHz). The reaction scope was explored using different reactants and the results of using this developed catalytic system demonstrated its capacity to reduce the reaction time and enhance the reaction efficiency to provide good to excellent product yield. Conversely, the simple recycling and reusability of this catalyst for at least six times without any noticeable leaching of copper makes it a potential future catalyst for synthesizing such compounds.

## Introduction

Green chemistry, also known as sustainable chemistry, is a sub-discipline of chemistry and is closely related to organic chemistry. It seeks to design chemical products and processes that minimize the use and generation of hazardous substances^1,2^. In the context of organic chemistry, green chemistry promotes the use of safe, sustainable, and economically viable methods in the synthesis of organic compounds. This includes the use of renewable feedstocks, the reduction or elimination of solvents or the use of safer alternatives, energy-efficient processes, the design of chemicals that are fully functional yet degrade into innocuous products after use, and utilizing catalytic substrates that are recyclable and reusable. The ultimate goal of green chemistry is to create better, safer chemicals while protecting and enhancing the Earth’s ecosystems. It’s a step towards a more sustainable and environmentally friendly chemical industry^3–6^.

In organic chemistry, heterogeneous recyclable catalytic substrates such as Fe_3_O_4_, SiO_2_, and MCM-41 play a pivotal role in facilitating chemical reactions. They provide a platform where reactants can come together to react more efficiently^7,8^. The use of catalytic substrates can significantly enhance the rate of reaction by lowering the activation energy, thereby making the reaction more feasible under mild conditions. This is particularly important in industrial applications where high temperatures and pressures can be costly and potentially hazardous^9,10^. the applications of catalytic substrates in organic chemistry are vast and varied. They are extensively used in the synthesis of pharmaceuticals, where they can help in the efficient production of complex organic compounds. they also find applications in the production of fine chemicals, in environmental chemistry where they are used in the breakdown of pollutants, and hydrogenation reactions, organocatalysts are used in the hydrogenation of organic unsaturated substrates^11–13^. Among these catalytic substrates, Fe_3_O_4_, or magnetite, is a versatile heterogeneous catalyst used in various reactions due to its unique properties like magnetism, recycling, and high thermal stability, and also, its surface can be easily modified, and enhancing its catalytic activity. Nowadays, Fe_3_O_4_ is used in various fields such as coupling reactions, environmental remediation, electrocatalysis, organic synthesis, biosensing, and drug delivery^14–17^.

Next, Click chemistry, a term coined by K. B. Sharpless in 2001, refers to a class of reactions that are high yielding, wide in scope, create only byproducts that can be removed without chromatography, are stereospecific, simple to perform, and can be conducted in easily removable or benign solvents^18,19^. The concept was further developed by Barry Sharpless at Scripps Research and Morten Meldal at the University of Copenhagen, who independently discovered a pivotal reaction that could link two molecules; an azide and an alkyne, with relative ease. Their groundbreaking work in this field earned them the Nobel Prize in chemistry^20,21^. The azide-alkyne cycloaddition, a cornerstone of click chemistry, involves a 1,3-dipolar cycloaddition between an azide and an alkyne to form a 1,2,3-triazole. The thermal Huisgen 1,3-dipolar cycloaddition requires elevated temperatures and often results in mixtures of regioisomers when asymmetric alkynes are used. However, the introduction of copper-catalyzed and ruthenium-catalyzed variants of this reaction has revolutionized the field. The copper-catalyzed reaction can be conducted under aqueous conditions at room temperature and specifically synthesizes 1,4-disubstituted regioisomers^22,23^. These catalyzed reactions have brought the azide-alkyne cycloaddition to the forefront as a prototype click reaction. The reaction of azide-alkyne cycloaddition, particularly the Copper(I)-catalyzed azide–alkyne cycloaddition (CuAAC), employs copper(I) as a catalyst. The reaction can be carried out using commercially available sources of copper(I) like cuprous bromide or iodide. Since Cu(I) is not stable in water-based solvents, it is more effective to use a combination of copper(II) (e.g., copper(II) sulfate) and a reducing agent (e.g., sodium ascorbate) to generate Cu(I) in situ^24–26^. The use of heterogeneous catalysts, such as those based on copper salts immobilized on inorganic solid supports, carbon, or magnetic materials, has also been explored. These catalysts can often be easily separated from the reaction mixture and reused, making them attractive for industrial applications^27,28^. While the use of Fe_3_O_4_ as a catalytic substrate in azide-alkyne cycloaddition reactions is less common, ongoing research continues to expand the range of catalytic substrates used in these reactions^29,30^.

Another application of recyclable catalytic substrates is their use in coupling reactions such as the Ullmann reaction. The Ullmann reaction, named after Fritz Ullmann, is a method for coupling two aryl or alkyl groups with the help of copper. This reaction was initially documented by Ullmann and his student Bielecki in 1901. It was among the pioneer reactions to employ a transition metal, primarily copper, in its higher oxidation states. Despite facing many obstacles in its early phases, the Ullmann reaction has garnered new interest in modern times because of the numerous advantages of copper over other catalytic metals for the synthesis of widely used biaryl structures^31–33^. Biaryls are commonly utilized as chiral phosphine ligands^34^, monomers of conductive polymers^35^, and as primary intermediates in the preparation of pharmaceutic drugs and natural products^36,37^. The traditional version of the Ullmann reaction requires harsh reaction conditions, which are carried out at high temperatures, and it has a reputation for reacting with disordered yields. On the other hand, these reactions mostly needed the use of large quantities of copper reagents, such as copper metal powder or copper(I) salts, which cause waste^38^. Subsequently, palladium-catalyzed homocoupling reactions were introduced to improve efficiency. In this method, due to the need for reducing agents and complex reagents, and the high cost of palladium metal, its large-scale and industrial applications were limited^39–41^. In the past decades, a huge amount of research works has been done on the Ullmann reaction, and many catalysts have been developed and processed to optimize its conditions, which were mainly based on Cu(I) and palladium^42–45^. Meanwhile, for the first time, Qiang Wu reported the use of a Cu(II)-based catalyst for the Ullmann reaction, which proved that Cu(II) can also be used as a catalyst in this type of reaction^46^.

**Figure 1:**
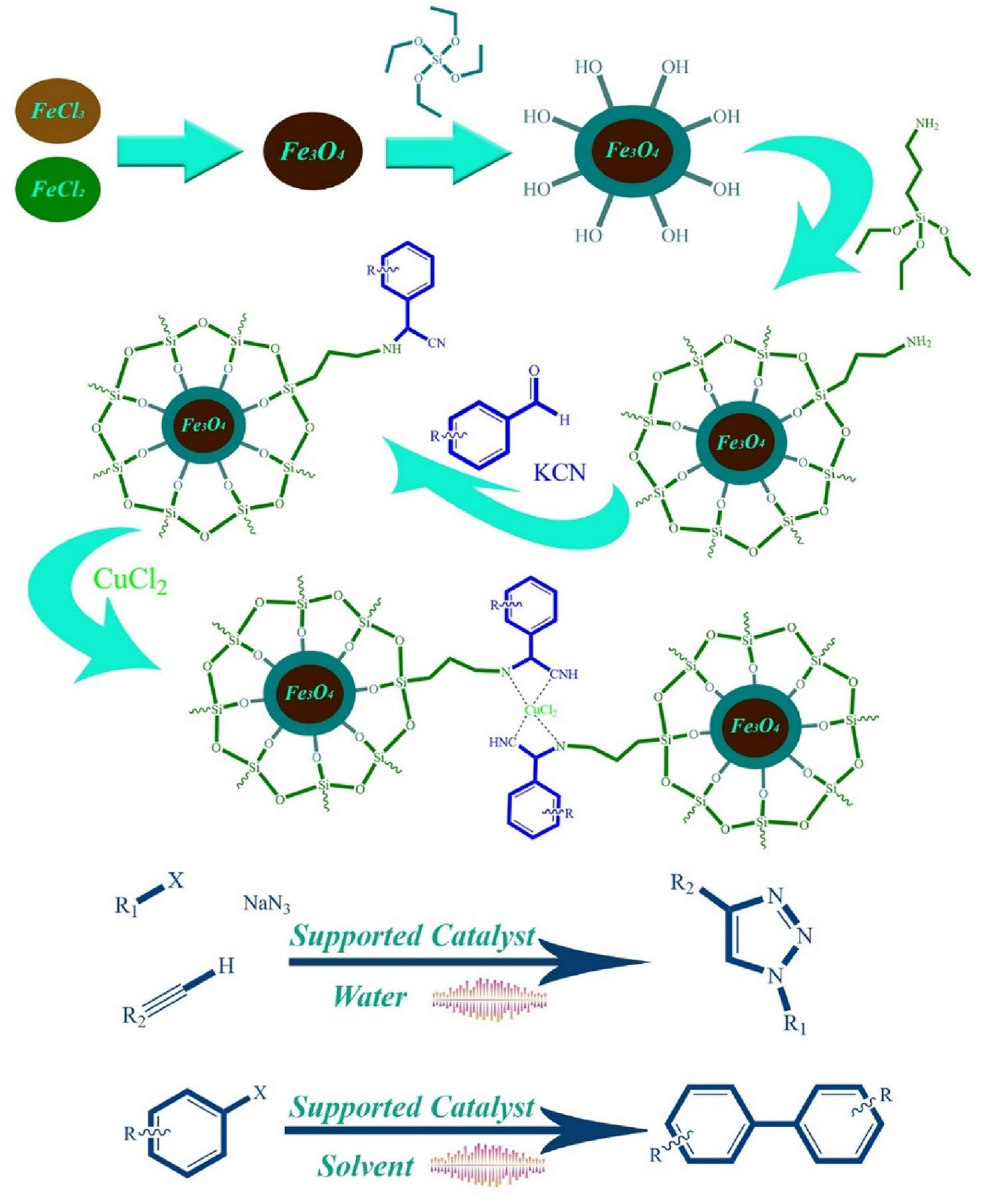
Catalyst synthesis procedure and its catalytic applications.

**Figure 2:**
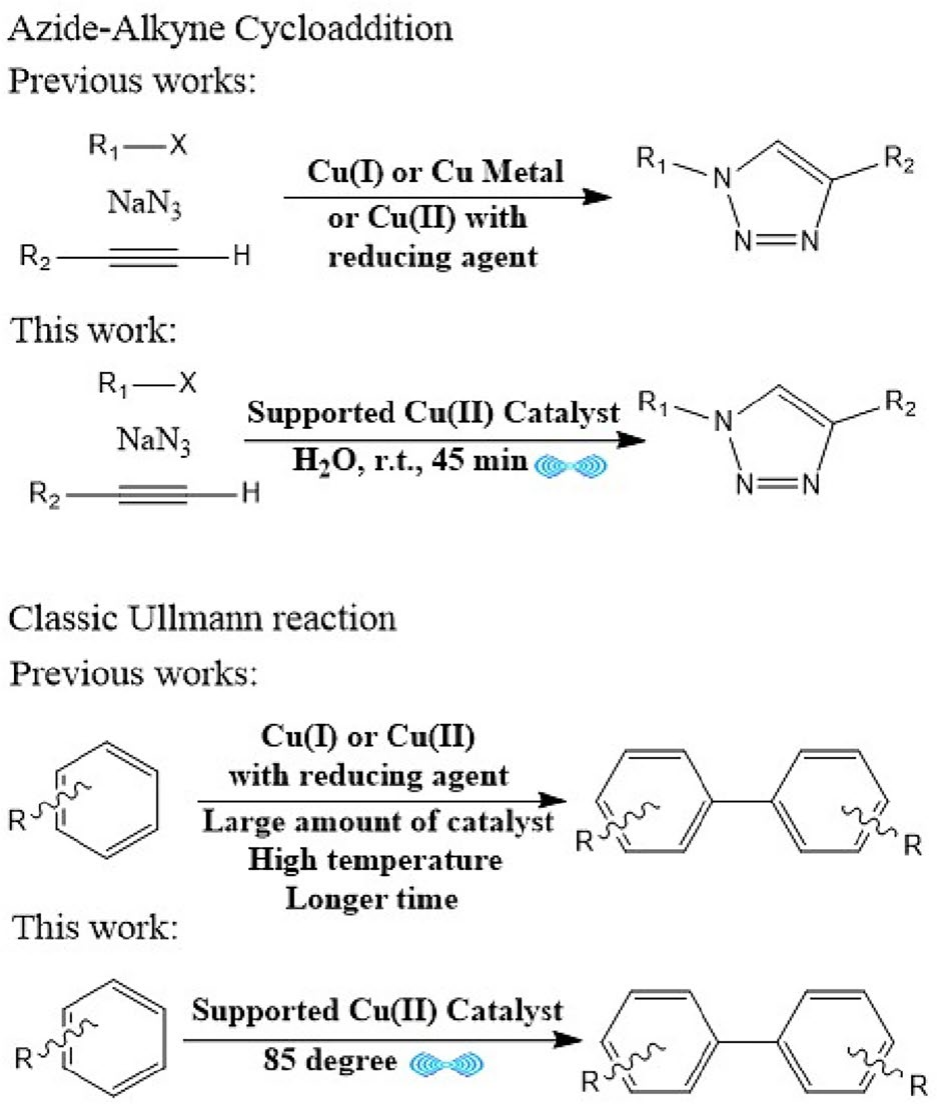
Comparison of previous reports with this work.

By considering previous reports and the need to simplify the conditions of these reactions as well as the use of cheaper and more stable catalysts, herein, for the first time, we developed the use of the Strecker reaction as a suitable approach for the synthesis of α-aminonitriles functional groups on the surface of the catalyst, leading to a unique ligand that has a high tendency to complexation with copper(II) nanoparticles to produce an easily accessible and cheap catalyst. In order to create the property of reusability and simple separation for the catalyst, an internal magnet core was considered (Fig. 1). This fabricated copper(II) catalyst used for ultrasound promoted synthesis of 1,4-disubstituted 1,2,3-triazoles and biaryls through azide–alkyne cycloaddition and Classic Ullmann reaction respectively. As mentioned, a simple copper(II) salt is employed for the preparation of the catalyst rather than using unstable copper(I) salts or copper metal, and the reaction conditions described in this article are milder compared to earlier findings, with a relatively lower quantity of catalyst being utilized without any reducing agent (Fig. 2).

## Experimental

### General

All reagents were purchased from Merck and Aldrich and used without further purification. Fourier transform infrared (FT-IR) spectra were made in KBr pellets on a Shimadzu IR470 spectrometer. Field emission scanning electron microscopy (FESEM) and energy dispersive x-ray (EDX) observations were carried out using a scanning electron microscope (TESCAN MIRA3). The amount of Cu(II) was estimated using an inductively coupled plasma optical emission spectrometer (ICP-OES; Perkin-Elmer 5300 DV). The magnetic property was investigated by a vibrating sample magnetometer (VSM, MDKB). Transmission Electron Microscopy (TEM) imaging was performed by using a Phillips CM120 TEM instrument. Thermogravimetric analysis (TGA) was carried out using a simultaneous thermal analyzer (Linseis STA PT 1600) at a heating rate of 10 °C/min under a nitrogen atmosphere. ^1^H NMR spectra were recorded with a Bruker DRX-400 AVANCE (Bruker, Ettlingen, Germany) instrument (400.1 MHz for ^1^H). NMR spectrums were taken in (DMSO-*d*_*6*_) solution and reported as parts per million (ppm).

### Preparation of the Fe_3_O_4_ NP

Fe_3_O_4_ in this study was prepared as described in the literature^47^, that FeCl_2_.4H_2_O (5.16 g) and FeCl_3_ 0.6H_2_O (14.16 g) were dissolved in the 300 mL degassed water with a magnet stirring, afterward, 30 mL of Concentrated ammonium hydroxide solution dropwise added to the mixture with simultaneous stirring for 30 min under the nitrogen gas atmosphere. Nanoparticles obtained in this step were collected using an external magnet, rinsed with deionized water perfectly until pH = 7, and dried under vacuum Desiccators.

### Preparation of the Fe_3_O_4_@SiO_2_

5.0 g of previously prepared Fe_3_O_4_ NPs were added to a round bottom balloon containing 50 mL deionized water and 200 mL ethanol and ultrasonically dispersed for 30 min. To the resulting suspension 2.5 mL of tetraethyl orthosilicate (TEOS) and 10 mL Concentrated NH_4_OH were added and stirred for 6 h at 50 °C. Then, filtered and washed with water and ethanol subsequently and dried under vacuum to obtain Fe_3_O_4_@SiO_2_.

### Preparation of the Fe_3_O_4_@SiO_2_-NH_2_

5.0 g of obtained Fe_3_O_4_@SiO_2_ NPs were dispersed in 50 ml of toluene and 50 ml of methanol, then ultrasonicated for 15 min and 5 ml of (3-aminopropyl) triethoxysilane (APTES) was added, stirred for 6 h. The product was collected and thoroughly washed with ethanol and dried under vacuum at 50 °C.

### Preparation of the Strecker-functionalized Fe_3_O_4_@SiO_2_

5.0 g of Fe_3_O_4_@SiO_2_-NH_2_ were dispersed in 30 ml of ethanol by ultrasonic for 15 min, then 0.42 g (4 mmol) benzaldehyde was added and stirred for 30 min at room temperature, 0.27 g (4 mmol) KCN was added to the mixture and continued to stir for 5 h, then, filtered and washed with ethanol and dried under vacuum to obtain.

### Preparation of the Cu(II)-α-aminonitrile-*f*-Fe_3_O_4_@SiO_2_ NP

2.0 g α-aminonitrile-*f*-Fe_3_O_4_@SiO_2_ was dispersed in water, and ultrasonication was performed at ambient temperature. Then, a solution of 0.17 g (1 mmol) CuCl_2_.2H_2_O in 10 mL of water was added dropwise to the reaction mixture, and for 12 h, The mixture was stirred at 40 °C. The obtained catalyst was then collected with an external magnet, washed three times with water and ethanol, and then dried under reduced pressure.

### General procedure for the catalytic azide–alkyne cycloaddition

Alkyne (1 mmol), alkylhalide (1 mmol), sodium azide (1.2 mmol) and Cu(II)-α-aminonitrile-*f*-Fe_3_O_4_@SiO_2_ NP (0.004 g, 0.1 mol% of Cu(II)) as a catalyst were added to 10 ml water and sonicated in ultrasonic apparatus at room temperature. The reaction progress was monitored by TLC (1:1 hexane: ethyl acetate, the chromatograms were visualized under UV 254–336 nm or iodine tank). After completion of the reaction, the water layer decanted off, and the residue dissolved in ethanol. Then, the catalyst was filtered with a nylon membrane and washed thrice with ethanol (30 mL). The ethanol layer was removed by rotary evaporator and the solid product was precipitated. For further purification, the product was crystallized in ethanol to obtain the desired pure products. The products were identified and confirmed using ^1^H NMR, melting point, and FT-IR, and then, the spectral data obtained were compared with credible references.

#### 1-benzyl-4-phenyl-1*H*-1,2,3-triazole (4a)

White solid, m.p. = 125–128 °C (Lit^48^. 128–130 °C); IR (KBr) Ѵ (cm^-1^): 3160 (C=C–H), 2953 (–C–H), 1580 (C = C), 1342 (C–N); ^1^H NMR (DMSO-d_6_, 400 MHz) δ (ppm): 5.37 (s, 2H, CH_2_), 7.21–7.26 (m, 3H, H_aromatic_), 7.51–7.60 (m, 5H, H_aromatic_), 7.73 (s, 1H, H_triazole_), 7.86 (d, J = 8.0 Hz, 2H, H_aromatic_); ^13^C NMR (DMSO*-d*_*6*_, 100 MHz) δ (ppm): 58.9, 117.1, 117.8, 123.0, 125.4, 128.8, 132.0, 137.8, 138.8, 139.7, 153.2.

### General procedure for the catalytic Ullmann Reaction

Under nitrogen gas, 5 mL of DMF, aryl halide (2 mmol), potassium fluoride (2 mmol), and Cu(II)-α-aminonitrile-*f*-Fe_3_O_4_@SiO_2_ NP (15 mol%) were added in a dry round-bottomed flask and the mixture was refluxed at 85 °C for 8 h under ultrasonication. The reaction progress was monitored by TLC (2:1 hexane: ethyl acetate), the chromatograms were visualized under UV 254–336 nm or iodine tank). After completion of the reaction, and then cooling, the solution was filtered under reduced pressure with a nylon membrane to separate the catalyst and washed with 10 ml of ethanol. The filtered solution was dried using sodium sulfate, filtered again, and then the solvent was removed with a rotary evaporator. The obtained product was recrystallized using ethyl acetate: ethanol (1:1) for further purification.

#### Biphenyl (6a)

White solid, m.p. 66–68 °C (Lit.^49^. 68–69 °C); IR (KBr) Ѵ (cm^-1^): 3052 (C=C–H, stretch), 1499 (C=C_aromatic_), 719 and 684 (C=C–H, bending); ^1^H NMR (DMSO-d_6_, 400 MHz) δ (ppm): 7.28 (t, J = 7.6 Hz, 2H, H_aromatic_), 7.51 (t, J = 7.6 Hz, 4H, H_aromatic_), 7.73 (d, J = 7.6 Hz, 4H, H_aromatic_).

## Results and discussion

### Preparation of catalyst

The modified catalyst synthesis method is shown in Fig. 1. At first, the magnetic core was formed using FeCl_2_.4H_2_O and FeCl_3_.6H_2_O. Then Fe_3_O_4_@SiO_2_ was produced by reacting the obtained product with TEOS in the mixture of water and ethanol as solvent at a temperature of 50 °C for 6 h. In the next step, Fe_3_O_4_@SiO_2_ was reacted with APTES in methanol and toluene for 6 h to embed the amine group and obtain Fe_3_O_4_@SiO_2_-NH_2_. By using the Strecker aminonitrile synthesis method, the previous step amino functionalized core was treated with benzaldehyde and potassium cyanide in ethanol to achieve α-aminonitrile-*f*-Fe_3_O_4_@SiO_2_. In the final step, by reacting with CuCl_2_.2H_2_O in water at 40 °C for 12 h, the ultimate catalyst was prepared. The synthesized catalyst was characterized using multiple methods.

### Characterization of the catalyst

#### FT-IR analysis

**Figure 3:**
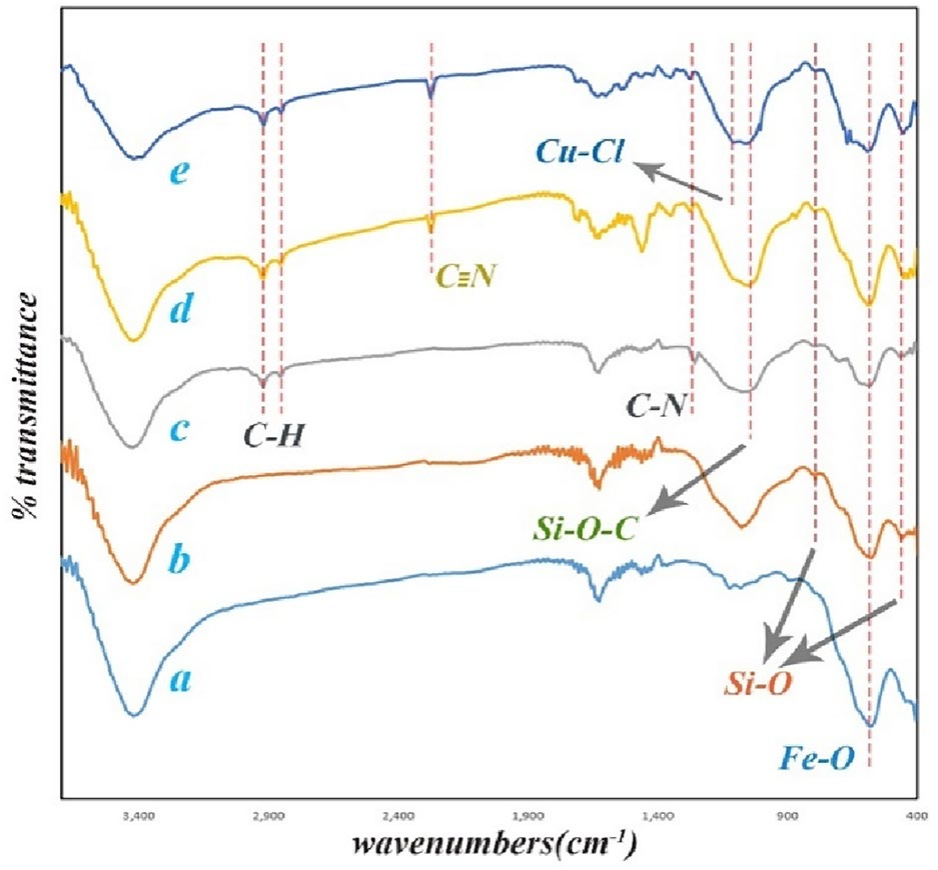
The FT-IR spectrum of all stages of the catalyst synthesis; (**a**) Fe_3_O_4_ NP (**b**) Fe_3_O_4_@SiO_2_ (**c**) Fe_3_O_4_@SiO_2_-NH_2_ (**d**) α-aminonitrile-*f*-Fe_3_O_4_@SiO_2_ (**e**) Cu(II)-α-aminonitrile-*f*-Fe_3_O_4_@SiO_2_.

FT-IR spectrums of all steps are shown in Fig. 3. In the FT-IR spectrum of Fe_3_O_4_, the peak at 582 cm^-1^ is related to Fe–O vibration, in the Fe_3_O_4_@SiO_2_ spectrum 460 cm^-1^ peak is corresponded to Si–O–Si vibration, and 792 and 1076 cm^-1^ peaks is related to unsymmetric and symmetric linear stretching. In Fe_3_O_4_@SiO_2_-NH_2_ spectrum, the peak at 1056 cm^-1^ is related to Si–O–C bond, the peak at 1257 cm^-1^ is relevant to C–N stretching and the peaks at 2850 and 2917 cm^-1^ is corresponded to C–H stretching. A significant peak for α-aminonitrile-*f*-Fe_3_O_4_@SiO_2_ appeared at 2268 cm^-1^ related to the nitrile group. In the final catalyst, an additional peak appeared at 1104 cm^-1^ for the Cu–Cl bond, indicating the presence of CuCl_2_ in this structure.

#### EDX and ICP-OES elemental analysis

**Figure 4:**
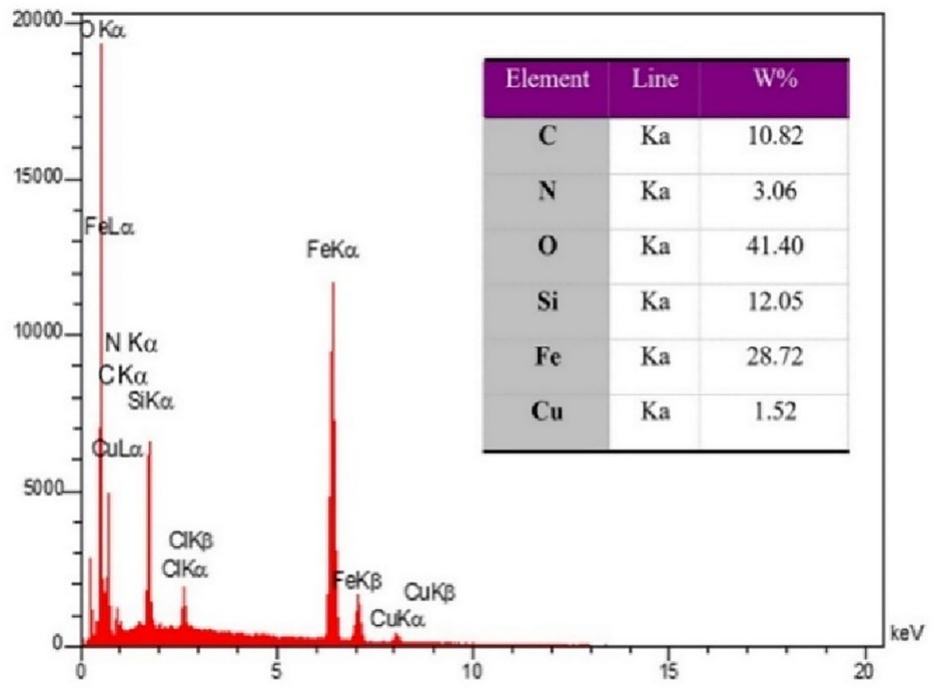
EDX spectroscopy of synthesized catalyst and resulting elemental percentage.

As exhibited in Fig. 4, the elemental analysis of Cu(II)-α-aminonitrile-*f*-Fe_3_O_4_@SiO_2_ with EDX spectroscopy shows the presence of C, N, O, Si, Fe, and Cu with the weight of 10.82, 3.06, 41.4, 12.05, 28.72, and 1.52% respectively, that matches with our suggested structure. For further validation, the percentage of Cu in the synthesized catalyst was examined by ICP-OES, which approved the EDX outcome with the result of 1.61% Cu.

#### VSM analysis

**Figure 5:**
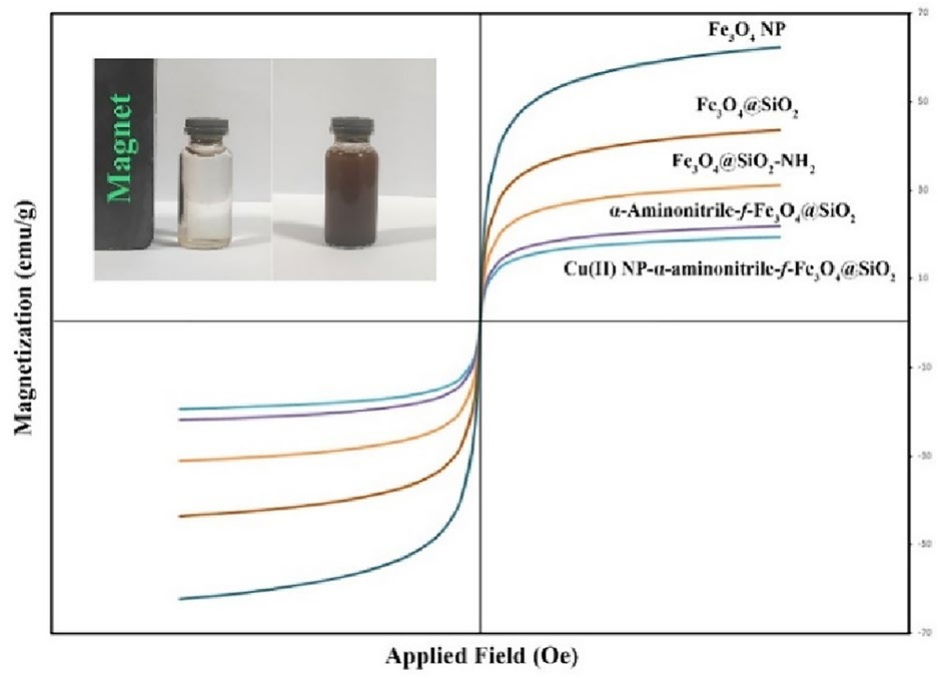
VSM spectrum of all steps of prepared catalyst.

The magnetic properties of all steps of the prepared catalyst were investigated, and the result is presented in Fig. 5. The magnetization saturation (Ms) values were recorded at 62.3, 43.6, 31.1, 21.8 and 19.4 emu/g for Fe_3_O_4_ NP, Fe_3_O_4_@SiO_2_, Fe_3_O_4_@SiO_2_-NH_2_, α-aminonitrile-*f*-Fe_3_O_4_@SiO_2_ and Cu(II)-α-aminonitrile-*f*-Fe_3_O_4_@SiO_2_ respectively. As expected, in each step of the reaction, due to the placement of organic layers and non-magnetic groups on the Fe_3_O_4_ core, its magnetic property decreased. Nevertheless, the final catalyst showed suitable magnetic properties, which can be used for easy separation of the catalyst in the reaction.

#### FESEM and TEM analysis

**Figure 6:**
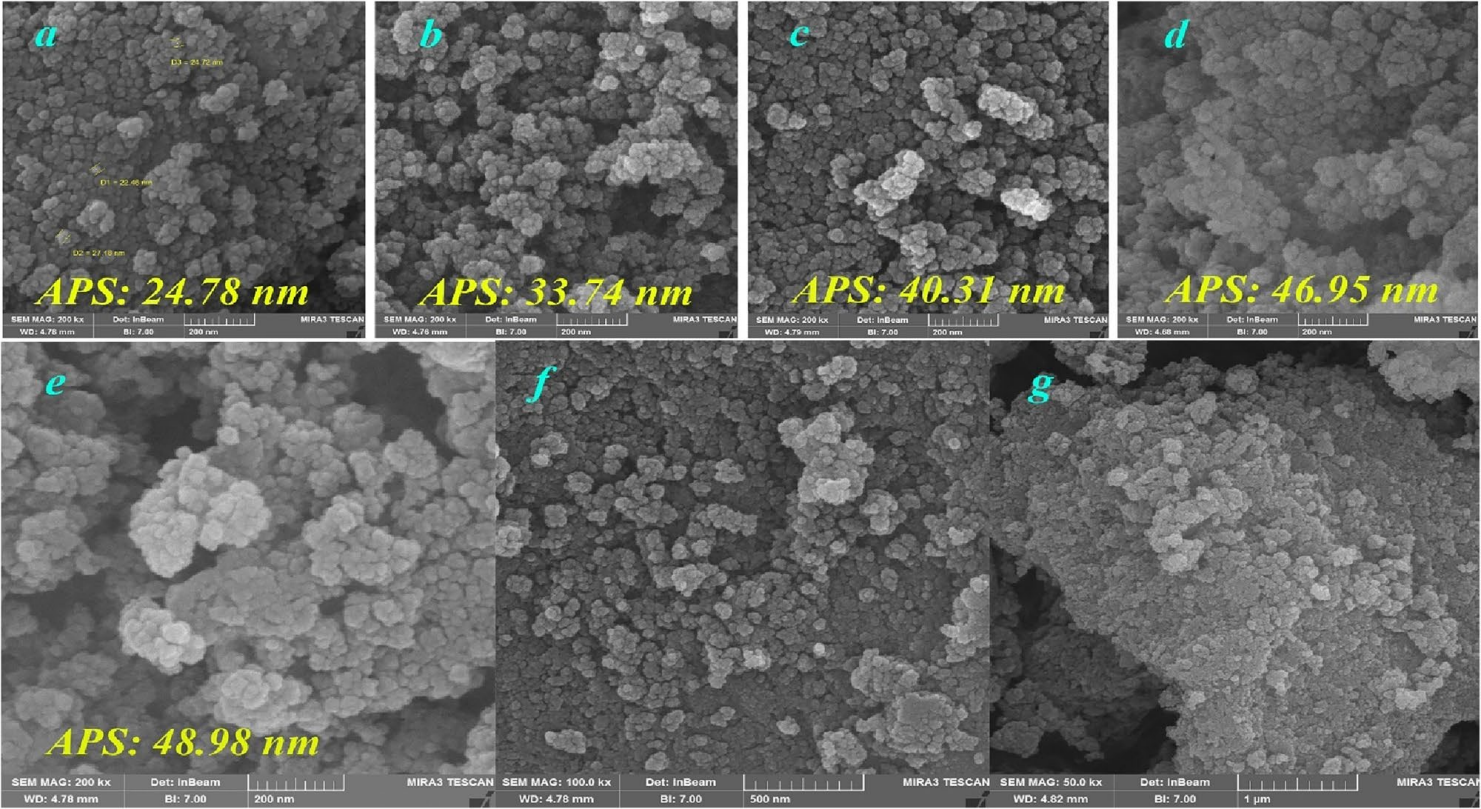
FESEM images of synthesized catalyst; (**a**) Fe_3_O_4_ NP (**b**) Fe_3_O_4_@SiO_2_ (**c**) Fe_3_O_4_@SiO_2_-NH_2_ (**d**) α-aminonitrile-f-Fe_3_O_4_@SiO_2_ (**e**) Cu(II)-α-aminonitrile-f-Fe_3_O_4_@SiO_2_ 200 nm (**f**) 500 nm (**g**) 1000 nm.

**Figure 7:**
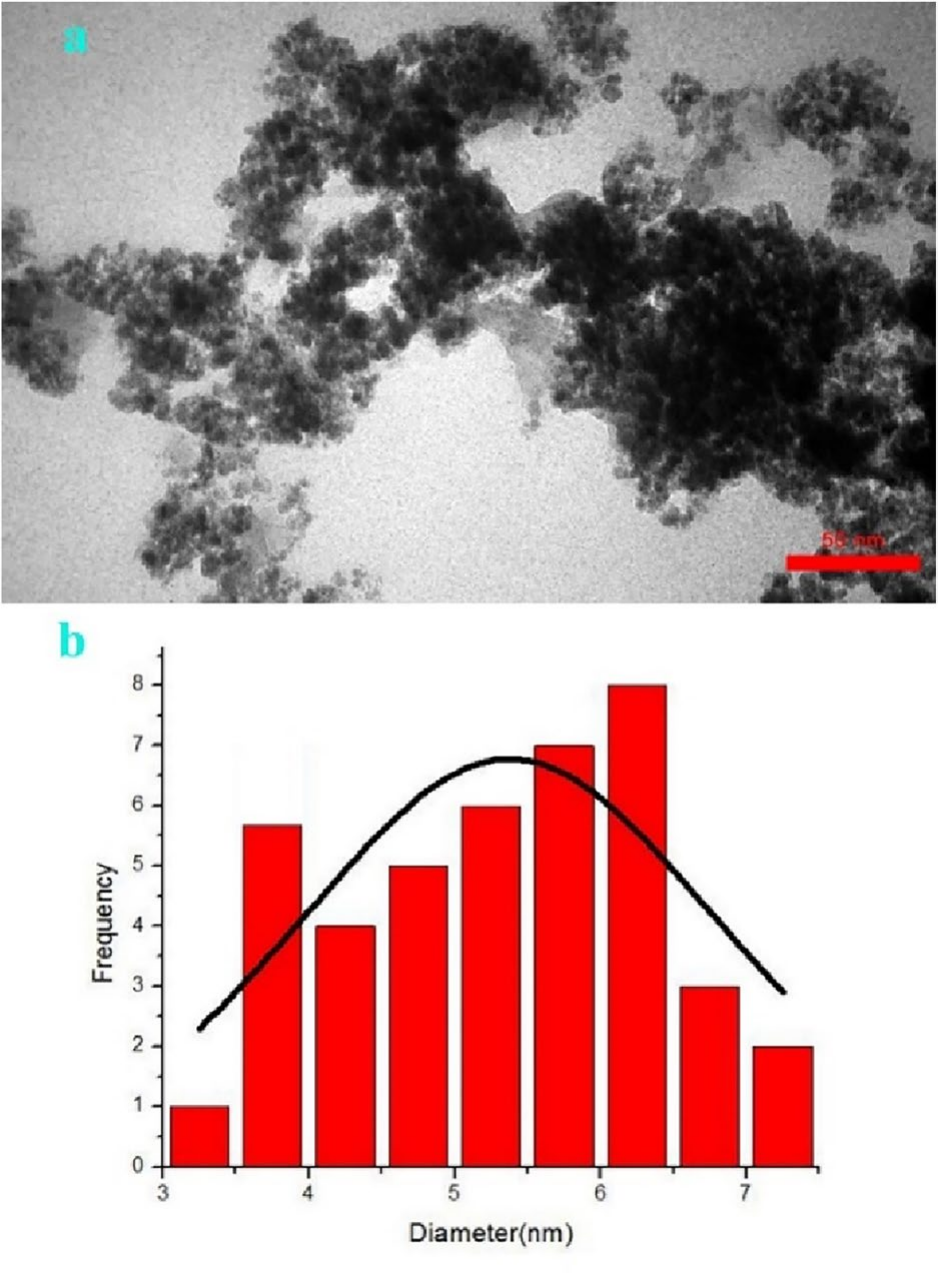
(**a**) TEM image of Cu(II)-α-aminonitrile-*f*-Fe_3_O_4_@SiO_2_, (**b**) TEM particle size distribution of Cu(II)-α-aminonitrile-*f*-Fe_3_O_4_@SiO_2_.

The morphology and particle size of all prepared catalyst phases were investigated by FESEM and TEM analysis. As shown in Fig. 6, the initial Fe3O4 prepared had a spherical morphology with an average diameter of 24.74 nm, also, the average particle size has increased in each step and it is a proof of the layered structure of this catalyst that after four-step modification, the final catalyst kept its spherical structure. The TEM spectrum taken from the catalyst is shown in Fig. 7, which gives us a higher spatial resolution image of this fabricated spherical catalyst, and its particles size. In this figure, the histogram of particle size distribution is also shown, which has an average diameter of 5.3 nm.

#### TGA analysis

**Figure 8:**
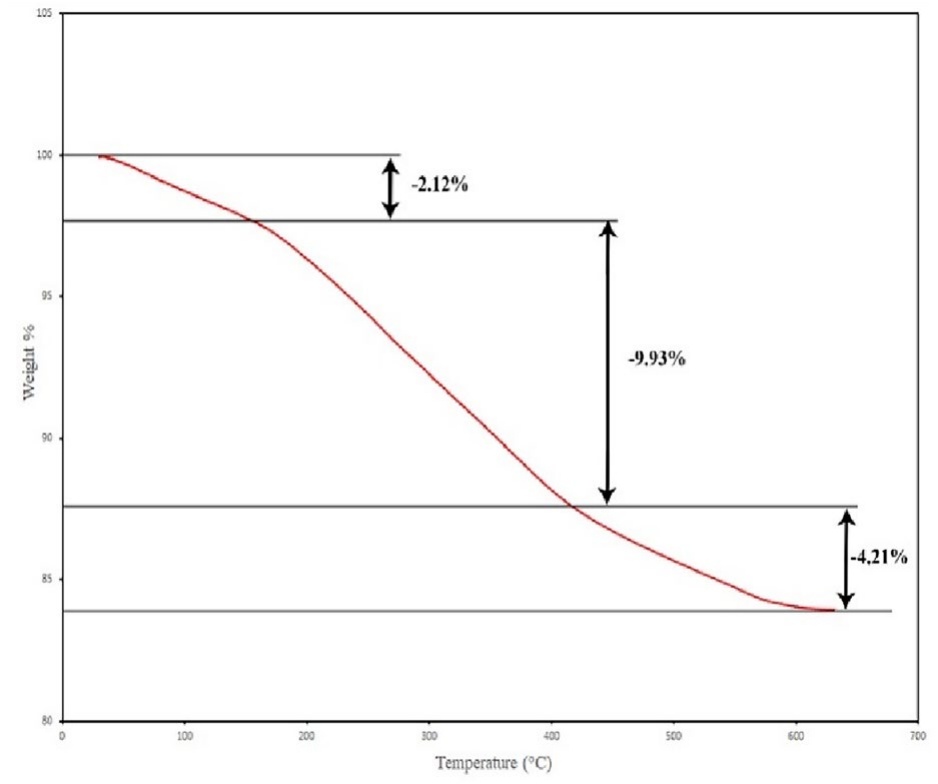
TGA analysis of Cu(II)-α-aminonitrile-*f*-Fe_3_O_4_@SiO_2_.

The thermal stability and properties of the fabricated catalyst were investigated by thermogravimetric analysis (TGA), and the result is reported in Fig. 8. The first decrease in mass is at a temperature below 170 °C, which is about 2.12% and is related to the loss of water. The second decrease in mass occurred at 170 to 430 °C, and the third decrease was at 430 to 630 °C, which was about 9.93% and 4.21%, respectively, which is related to the destruction of the organic segment of the synthesized catalyst, that, it is entirely consistent with the information obtained from the EDX spectrum of the catalyst.

### Investigation of catalyst activity

In order to investigate the catalytic effect of the prepared catalyst, it was used in the azide-alkyne cycloaddition reaction using the three-component reaction of sodium azide, alkyl halide and alkyne, as well as in the classical Ullmann coupling reaction using aryl halides, which produced products with a good yield up to Excellent result.

For the purpose of the azide-alkyne cycloaddition reaction conditions, the reaction of phenylacetylene, benzyl chloride and, sodium azide was investigated as a primary model. The reaction was performed in the presence of various amounts of catalyst in water as media, at room temperature, and it was found that the best result is obtained when the reaction is carried out in the presence of 0.1 mol% (0.004 g) catalyst (Table 1).

**Table 1:** Optimizing the amount of catalyst in the model reaction for the synthesis of 1-benzyl-4-phenyl-1*H*-1,2,3-triazole.^a^

Entry	Catalyst amount (mol%)	Time (min)	Yield (%)^b^
1	–	120	0
2	0.05	90	73
3	0.1	45	94
4	0.2	45	91
5	0.3	45	89
6	0.4	45	93

^a^Reaction conditions: phenylacetylene (1 mmol), sodium azide (1.2 mmol), benzyl chloride (1 mmol) and Cu(II)-α-aminonitrile-*f*-Fe_3_O_4_@SiO_2_ NP were charged to the H_2_O as solvent (10 mL) under ultrasonic irradiation at ambient temperature.

^b^Isolated yields.

In the investigation for improved efficiency, different solvents were used under different conditions in the reaction of phenylacetylene, benzylchloride and sodium azide with the optimal amount of Cu(II)-α-aminonitrile-*f*-Fe_3_O_4_@SiO_2_ as a model reaction. According to the results in Table 2, the presence of water as a co-solvent in the reaction is very important to increase the efficiency, and the best yield is obtained when the reaction is carried out in pure H_2_O as a solvent for 45 min at room temperature under ultrasonic irradiation (Table 2).

**Table 2:** Optimizing the reaction condition in the model reaction for the synthesis of 1-benzyl-4-phenyl-1*H*-1,2,3-triazole.^a^

Entry	Solvent	Temp (°C)	Time (min)	Yield (%)^b^
1	MeOH	60	120	Trace
2	EtOH	70	120	Trace
3	H_2_O:MeOH	25	60	65
4	H_2_O:MeOH	40	90	68
5	H_2_O:MeOH	60	90	74
6	H_2_O:EtOH	25	60	71
7	H_2_O:EtOH	50	60	75
8	H_2_O:EtOH	75	90	75
9	H_2_O:Acetone	25	90	25
10	H_2_O:Acetone	50	120	28
11	H_2_O	25	45	94
12	H_2_O	70	60	94

^a^ Reaction conditions: phenylacetylene (1 mmol), sodium azide (1.2 mmol), benzyl chloride (1 mmol) and Cu(II)-α-aminonitrile-*f*-Fe_3_O_4_@SiO_2_ NP (0.1 mol%) were charged to the solvent (10 mL) at the specified temperature under ultrasonic irradiation.

^b^ Isolated yields.

To determine the extent and limitation of this catalytic reaction, various aryl halides and acetylenes were used in the reaction with sodium azide in the presence of the catalyst, under ultrasound in optimal conditions (Table 3). By analyzing the obtained results, we come to the conclusion that bromide in benzyl bromides is a better leaving group than iodide and chloride, and also strong electron-withdrawing groups on benzyl halides mainly in the para position accelerate the reaction and increase the efficiency.

**Table 3:** Preparation of 1,2,3-triazoles from the reaction of aryl halides and acetylenes and sodium azide catalyzed by Cu(II)-α-aminonitrile-*f*-Fe_3_O_4_@SiO_2_.


Entry	Halide	Alkyne	Product	Yield^a^ (%)	Time (h)	m.p. (°C)
1				94	0.75	125–128 °C (Lit.^48^. 128–130 °C)
2				92	0.5	149–150 °C (Lit.^48^. 151–152 °C)
3				92	0.75	89–90 °C (Lit.^50^. 87–89 °C)
4				89	1	94–97 °C (Lit.^51^. 96–97 °C)
5				97	0.75	142–143 °C (Lit.^48^. 140–141 °C)
6				91	0.5	212–214 °C (Lit.^52^. 214–215 °C)
7				96	1	125–128 °C (Lit.^48^. 128–130 °C)
8				89	1	125–128 °C (Lit.^48^. 128–130 °C)
9				92	0.75	150–153 °C (Lit.^53^. 152–154 °C)
10				93	0.75	201–203 °C (Lit.^52^. 202–204 °C)
11				84	1.25	Oil (Lit.^51,54^)

^a^ Isolated yields.

In the continuation of this study, the catalytic effects of the fabricated catalyst were investigated through the Ullmann reaction. So that the optimization of the amount of catalyst was needed, the homo-coupling reaction of iodobenzene in DMF and potassium fluoride in the presence of catalyst was selected as a model reaction. As can be seen, the copper catalyst with the amount of 15 mol% gave us the best result with the highest yield, and using more amount of catalyst and spending more time did not affect the yield (Table 4).

**Table 4:** Optimizing the amount of catalyst in the model reaction for the synthesis of biphenyl.

Entry	Catalyst amount (mol%)	Time (h)	Yield (%)
1	–	24	0
2	5	12	63
3	10	8	80
4	15	8	93
5	20	8	93
6	20	12	93

^a^Reaction conditions: under nitrogen atmosphere, 5 ml of DMF, iodobenzene (2 mmol), potassium fluoride (2 mmol) and Cu(II)-α-aminonitrile-*f*-Fe_3_O_4_@SiO_2_ NP were added.

in a dry round-bottomed flask and the mixture was refluxed at 85 °C under ultrasonication.

^b^Isolated yields.

To find the best reaction conditions, the model reaction was performed in several conditions in the presence of the catalyst. As shown (Table 5), KF is a better base in presence of K_2_CO_3_, Cs_2_CO_3_, and KOH, and in addition, DMF and DMSO are more reasonable choices due to their high polarity and high heat capacity, and this issue is also known experimentally in the reaction optimization. Considering all these results, the reaction in DMF as solvent and potassium fluoride base at 85 °C for 8 h under ultrasound was selected as the model reaction.

**Table 5:** Optimizing the reaction condition in the model reaction for the sy.

Entry	Solvent	Base	Temp (°C)	Time (h)	Yield^b^ (%)
1	EtOH	K_2_CO_3_	78	12	Trace
2	EtOH	KF	78	12	Trace
3	Toluene	K_2_CO_3_	85	12	Trace
4	Toluene	KF	85	12	Trace
5	DMSO	K_2_CO_3_	85	12	68
6	DMSO	Cs_2_CO_3_	85	12	59
7	DMSO	KF	70	8	73
8	DMSO	KF	85	12	77
9	DMF	K_2_CO_3_	85	12	74
10	DMF	Cs_2_CO_3_	85	12	69
11	DMF	KOH	85	12	84
12	DMF	KF	85	8	93
13	DMF	KF	85	12	93

^a^Reaction conditions: under nitrogen atmosphere, 5 ml of solvent, iodobenzene (2 mmol), base (2 mmol) and, 15 mol% Cu(II)-α-aminonitrile-*f*-Fe_3_O_4_@SiO_2_ NP were added in a dry round-bottomed flask and the mixture was refluxed at the specified temperature for the mentioned time under ultrasonication.

^b^Isolated yields.

We generalized the use of this fabricated catalyst in the Ullmann reaction under optimal conditions using various aryliodides, chlorides, and bromides as the substrates and several alkynes; the results are summarized in Table 6. In this reaction, iodide and bromide are better leaving groups than chloride, and electron-withdrawing groups on aryl halides increase and electron-donating groups decrease efficiency.

**Table 6:** Preparation of biaryls from the reaction of aryl halides catalyzed by Cu(II) -α-aminonitrile-*f*-Fe_3_O_4_@SiO_2_.


Entry	Aryl Halide	Product	Yield^a^ (%)	Time (h)	m.p. (°C)
1			93	8	66–68 °C (Lit.^49^ 68–69 °C)
2			92	10	120–122 °C (Lit.^49^ 119–120 °C)
3			92	8	175–178 °C (Lit.^55^ 176–178 °C)
4			96	8	234–236 °C (Lit.^56^ 237–238 °C)
5			94	9	234–236 °C (Lit.^56^ 237–238 °C)
6			92	10	66–68 °C (Lit.^49^ 68–69 °C)
7			86	12	120–122 °C (Lit.^49^ 119–120 °C)
8			89	12	175–178 °C (Lit.^55^ 176–178 °C)
9			78	12	66–68 °C (Lit.^49^ 68–69 °C)
10			87	12	193–194 °C (Lit.^57^ 190–192 °C)

^a^Isolated yields.

A comparison of the method developed in this article has been made with previous reports. As shown in Table 7 (entries 1–7), in this work, the products synthesized in the presence of Cu(II)-α-aminonitrile-*f*-Fe_3_O_4_@SiO_2_ as a catalyst provide excellent yields under milder conditions than previously reported papers. Likewise, in the Ullmann reaction, the use of the designed catalyst in the reaction of aryl halides accelerated the reaction and increased the yield in some cases (Table 7, entries 8–12). in contrast, the previously reported works give the desired products in more challenging conditions or more extended time.

**Table 7:** Comparison of the present method with previous works.

Entry	Product	Time	Yield (%)	Ref
1		45 min	92	This work
2^a^		120 min	89	^58^
3		45 min	97	This work
4^b^		240 min	90	^59^
5		45 min	93	This work
6^c^		120 min	85	^60^
8		8 h	92	This work
9^d^		24 h	92	^61^
10		8 h	96	This work
11^e^		7 h	90	^62^
12^f^		20 h	96	^46^

Reaction conditions: ^a^benzyl chloride (1 mmol) and NaN_3_ (1.5 mmol) with phenylacetylene (1.2 mmol) and Cu-Cu_2_O@RGO (5 mol%) in water.

^b^phenylacetylene (0.5 mmol), NaN_3_ (0.55 mmol) and benzyl halide (0.55 mmol) with Cu/CeO_2_ in water at 70 °C.

^c^acetylene (1.0 mmol), NaN_3_ (1.0 mmol) and benzyl halide (1.0 mmol) with CuI (20 mol%) and Eosin Y (0.1 mol%) in ethanol:water under Compact fluorescent light irradiation.

^d^aryl halide (1 mmol), base (1.0 mmol), glucose (0.25 mmol), cyclodextrin RM-*β*-CD (100 mg), Pd/Fe_3_O_4_@PDA (50 mg) in water under reflux.

^e^aryl halide (1 mmol), Et_3_N (0.5 mmol), TiO_2_–AA–Pd (0.3 mol%) solvent free under reflux.

^f^Under N_2_, aryl halide (2.0 mmol), KF (2.0 mmol), silica-supported copper(II) catalyst (500 mg) in DMSO under reflux at 130 °C.

### Recycling of the catalyst

**Figure 9:**
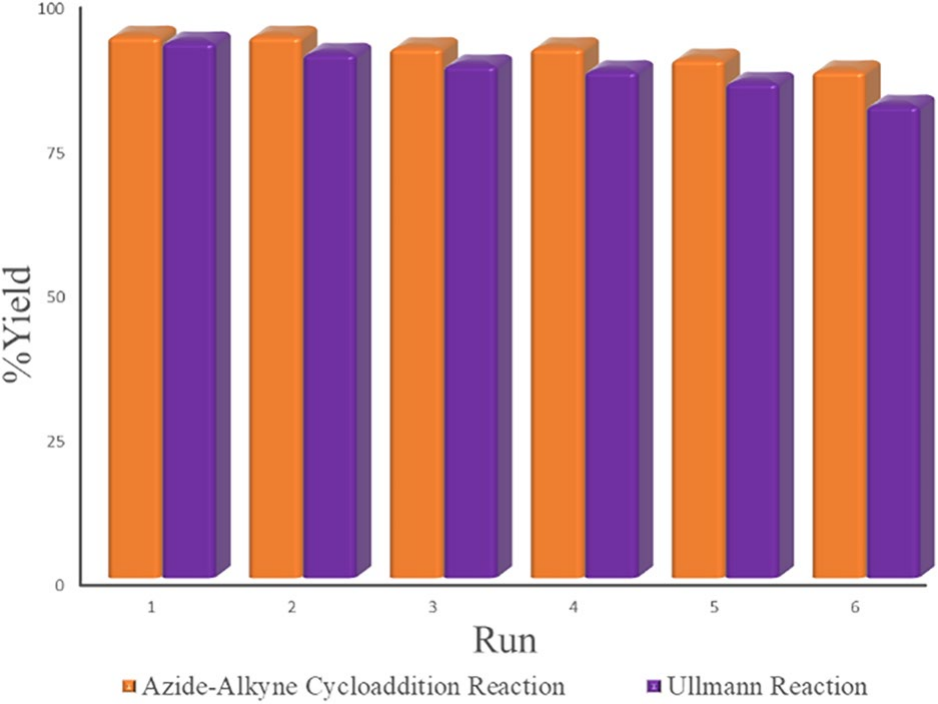
Reusability of the catalyst in the Azide–Alkyne Cycloaddition and Ullmann Coupling reaction.

For the practicable applications of such heterogeneous systems, the reusability of the catalyst is one of the essential properties. This feature was investigated using azide–alkyne cycloaddition and Ullmann Coupling model reactions under optimal conditions. Then, after each use, the catalyst was separated by simple filtration, thoroughly washed with water and ethanol, and dried. After six consecutive uses of the catalyst, the IR spectrum was taken, which did not show a significant change compared to the original catalyst. Based on the results obtained from the measurement of catalyst recovery (Fig. 9) and IR spectrum, this catalyst can be used for at least six cycles without any significant change in its catalytic activity.

### Proposed mechanism

**Figure 10:**
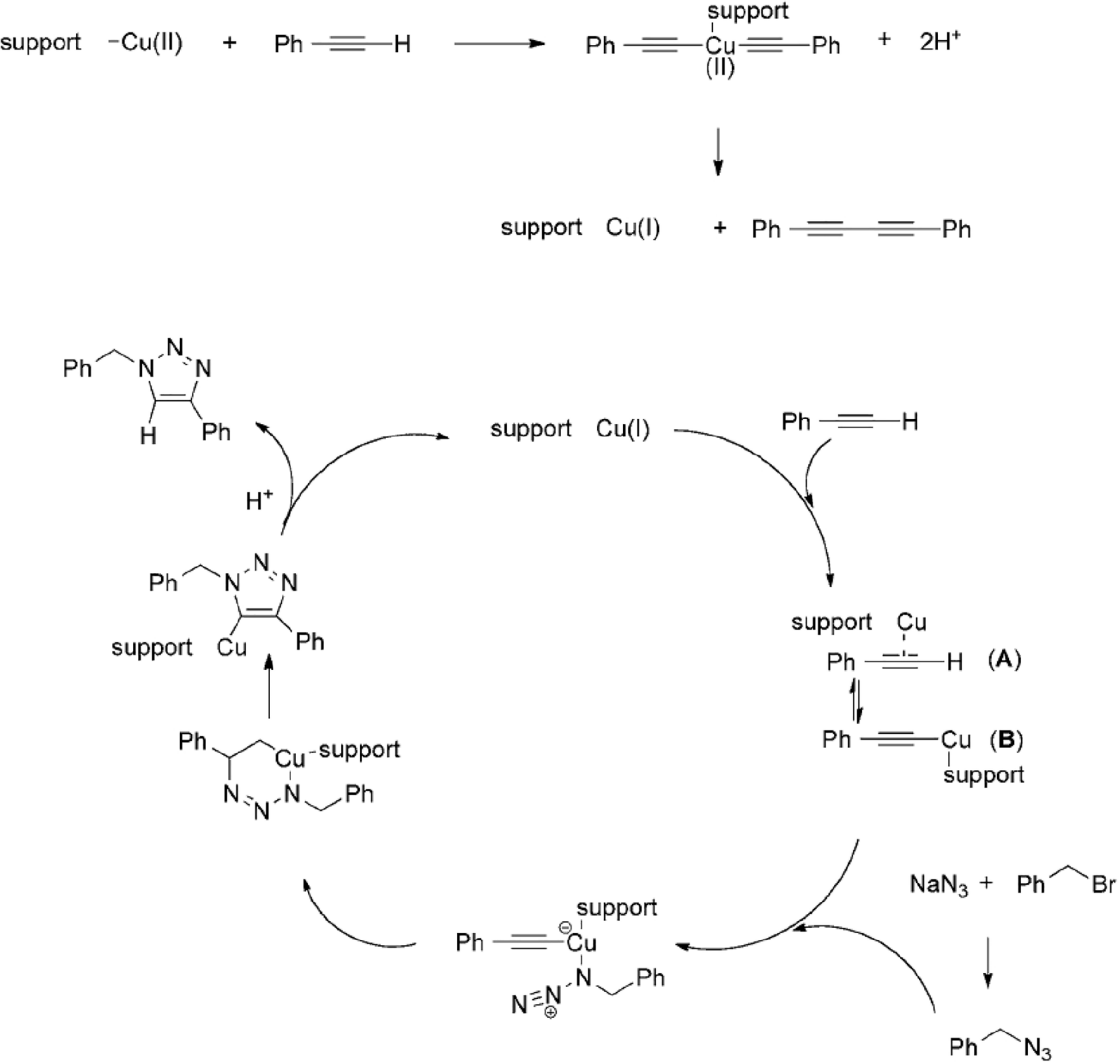
Proposed reaction mechanism for the supported Cu(II)-catalyzed Azide–Alkyne Cycloaddition reaction.

Based on previously published reports and investigations^63–65^, the suggested process for Azide-Alkyne Cycloaddition is illustrated in Fig. 10. In the first step, alkyne-alkyne homocoupling is performed on the Cu(II) substrate to form catalytically active Cu(I) species. Then, the cycloaddition reaction probably proceeds via the reaction mechanism proposed by Sharpless et al^66,67^.

**Figure 11:**
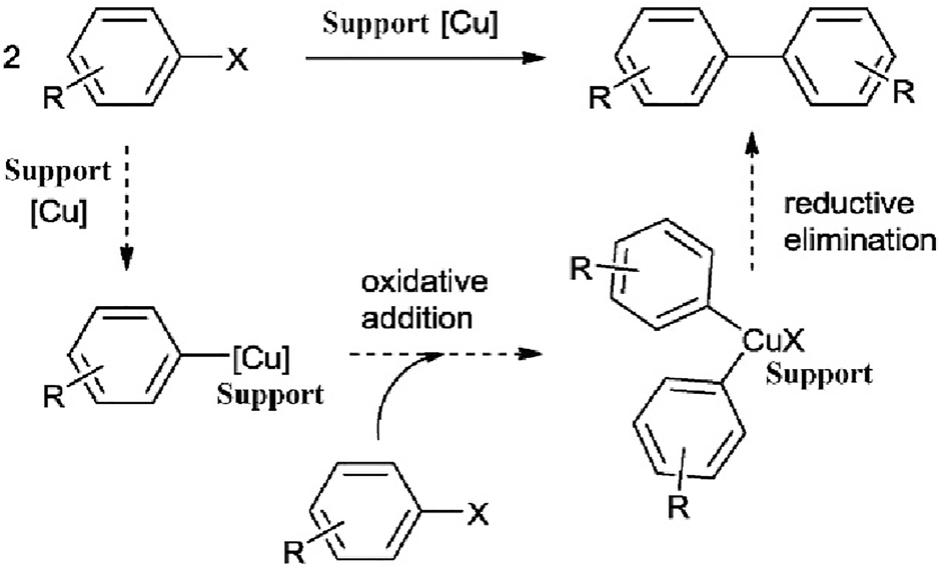
Proposed reaction mechanism for the supported Cu(II)-catalyzed Ullmann reaction.

Next, for the Ullmann reaction, there are several reports that confirm this reaction is carried out with copper(II)^46,68,69^, according to them and our limited knowledge at the molecular level, it is assumed that DMF in the presence of potassium fluoride partially reduces copper(II) and produces copper(I), which in the following, the Ullmann coupling reaction is performed using copper(I) (Fig. 11).

## Conclusion

In this article, for the first time, the Strecker multicomponent reaction has been used to modify the catalyst surface and prepare various functional groups in a straightforward step. Next, copper(II) metal was incorporated into the prepared ligand structure instead of unstable Cu(I) or Cu metal in older methods to make a novel Cu(II)-α-aminonitrile-*f*-Fe_3_O_4_@SiO_2_ NP catalyst. The structure of the prepared catalyst was also investigated using several methods, which entirely agree with the specified outline. This catalyst can be easily separated, recycled, and reused for at least six consecutive times. Cu(II)-α-aminonitrile-*f*-Fe_3_O_4_@SiO_2_ NP catalyst employed as an efficient catalyst with the aid of ultrasonication for the synthesis of 1,4-disubstituted 1,2,3-triazoles and biaryls via azide–alkyne Huisgen cycloaddition and Classic Ullmann reaction respectively. The applied methodology offers several advantages, such as the use of commercially available Cu(II) salts as an alternative to the traditional routine Cu metal or unstable Cu(I) catalysts, inexpensive precursors, catalyst simple preparation procedure, reusability and high activity for catalyst, also milder condition and excellent yields for the synthesis of triazole and biaryl derivatives. As far as we know, Strecker-functionalized catalysts have not been explored, which can be a starting point for further studies on this crucial method to prepare diverse and different ligand substrates and widely use them to make the demanded surfaces.

### Supplementary Information

Supplementary Information.

## Data Availability

All data generated or analysed during this study are included in this published article [and its supplementary information files].
