# Author Correction: Ultrasound-assisted Cu(II) Strecker-functionalized organocatalyst for green azide–alkyne cycloaddition and Ullmann reactions

**DOI:** 10.1038/s41598-024-68133-z

**Published:** 2024-08-09

**Authors:** Mahyar Aghajani, Minoo Dabiri

**Affiliations:** Department of Organic Chemistry and Oil, Faculty of Chemistry and Petroleum Sciences, Shahid Beheshti University, Tehran, 1983969411 Islamic Republic of Iran

Correction to: *Scientific Reports* 10.1038/s41598-024-62826-1, published online 27 May 2024

In the original version of this Article, Minoo Dabiri was omitted as a corresponding author. The correct corresponding authors for this Article are Mahyar Aghajani and Minoo Dabiri.

The original Article has been corrected.

