# Supplementary material for: Ultrasound-assisted Cu(II) Strecker-functionalized organocatalyst for green azide–alkyne cycloaddition and Ullmann reactions
Source: Sci Rep. 2024 May 27;14:12141. doi: 10.1038/s41598-024-62826-1 (PMC11130308; doi:10.1038/s41598-024-62826-1)
Supplement: Supplementary file 1 — Supplementary Information. [file 41598_2024_62826_MOESM1_ESM.docx]

Ultrasound-Assisted Cu(II) Strecker-functionalized Organocatalyst for Green Azide-Alkyne Cycloaddition and Ullmann Reactions

Mahyar Aghajani ^a,^* , Minoo Dabiri ^a^

^a^ Department of Organic Chemistry and Oil, Faculty of Chemistry and Petroleum Sciences, Shahid Beheshti University, Tehran, 1983969411, Islamic Republic of Iran

**Spectral data of synthesized compounds:**

**Azide–Alkyne Cycloaddition:**


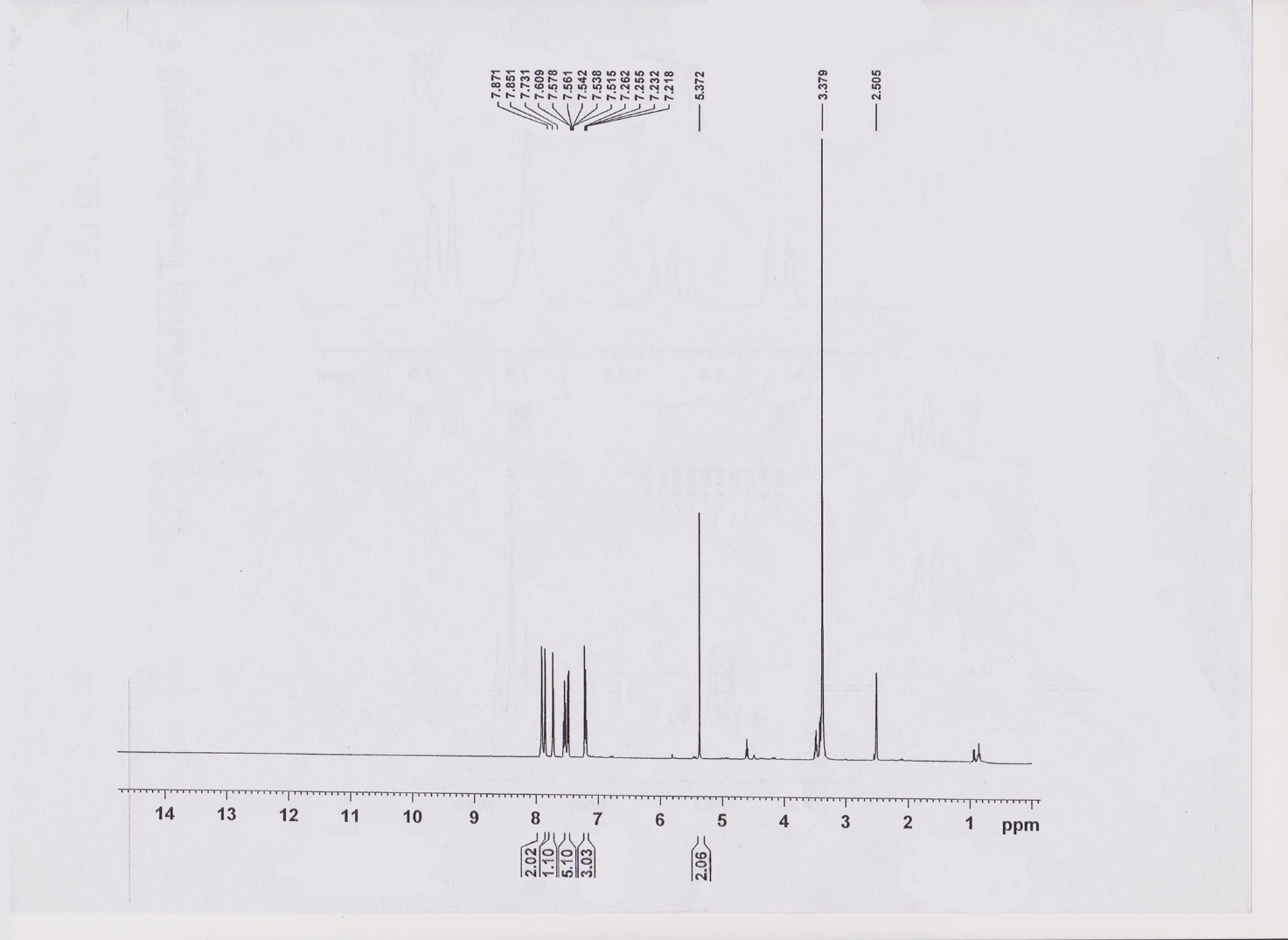


Figure 1. ^1^H NMR 1-Benzyl-4-phenyl-1H-1,2,3-triazole (4a)


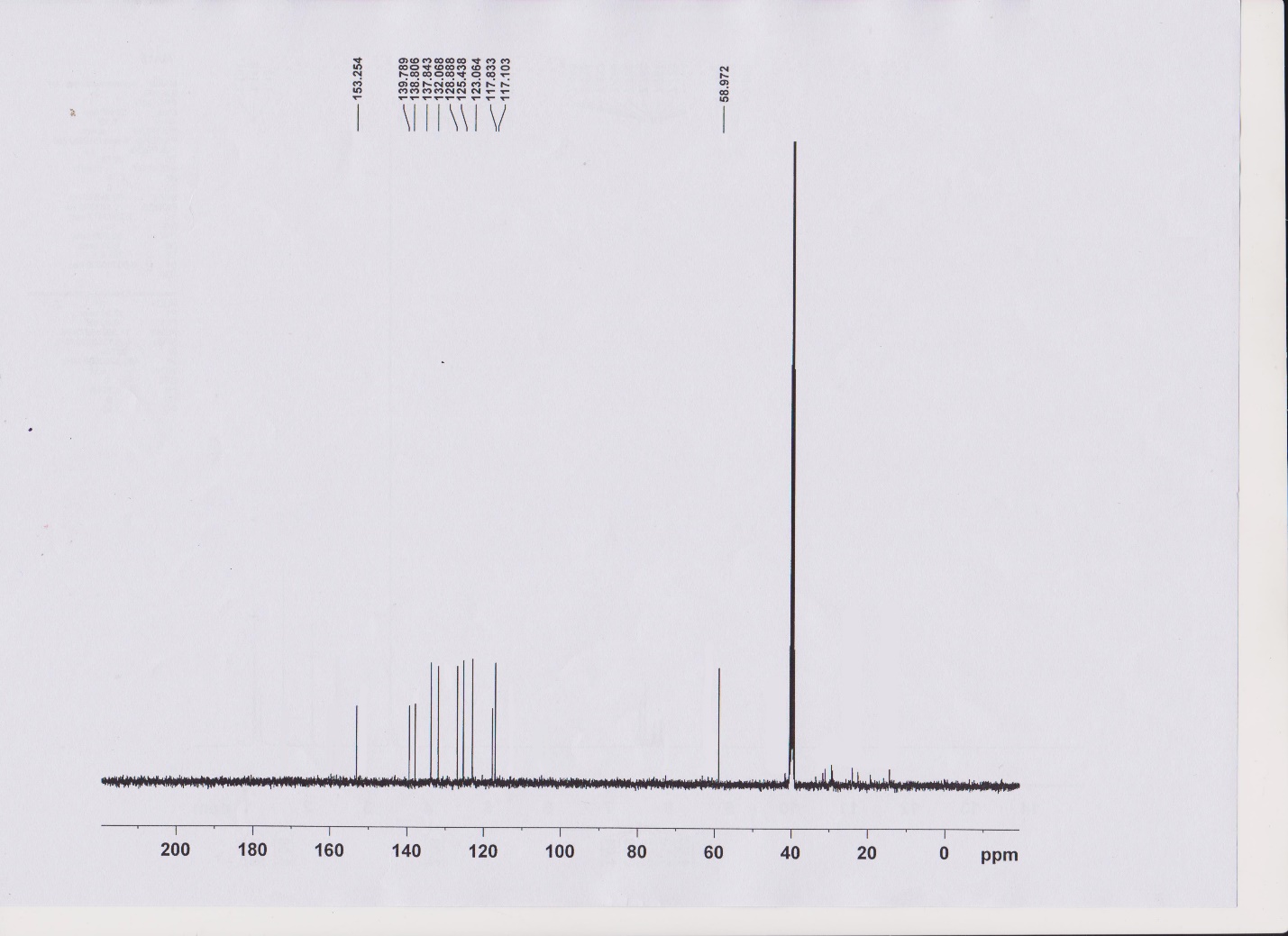


Figure 2. ^13^C NMR 1-Benzyl-4-phenyl-1H-1,2,3-triazole (4a)

2.7.1. 1-Benzyl-4-phenyl-1H-1,2,3-triazole (4a)

White solid, m.p. = 125-128 ºC (Lit. [1]. 128–130 ºC); IR (KBr) Ѵ (cm^-1^): 3160 (C=C–H), 2953 (–C–H), 1580 (C=C), 1342 (C–N); ^1^H NMR (DMSO-d_6_, 400 MHz) δ (ppm): 5.37 (s, 2H, CH_2_), 7.21–7.26 (m, 3H, H_aromatic_), 7.51–7.60 (m, 5H, H_aromatic_), 7.73 (s, 1H, H_triazole_), 7.86 (d, J = 8.0 Hz, 2H, H_aromatic_); ^13^C NMR (DMSO-d_6_, 100 MHz) δ (ppm): 58.9, 117.1, 117.8, 123.0, 125.4, 128.8, 132.0, 137.8, 138.8, 139.7, 153.2.


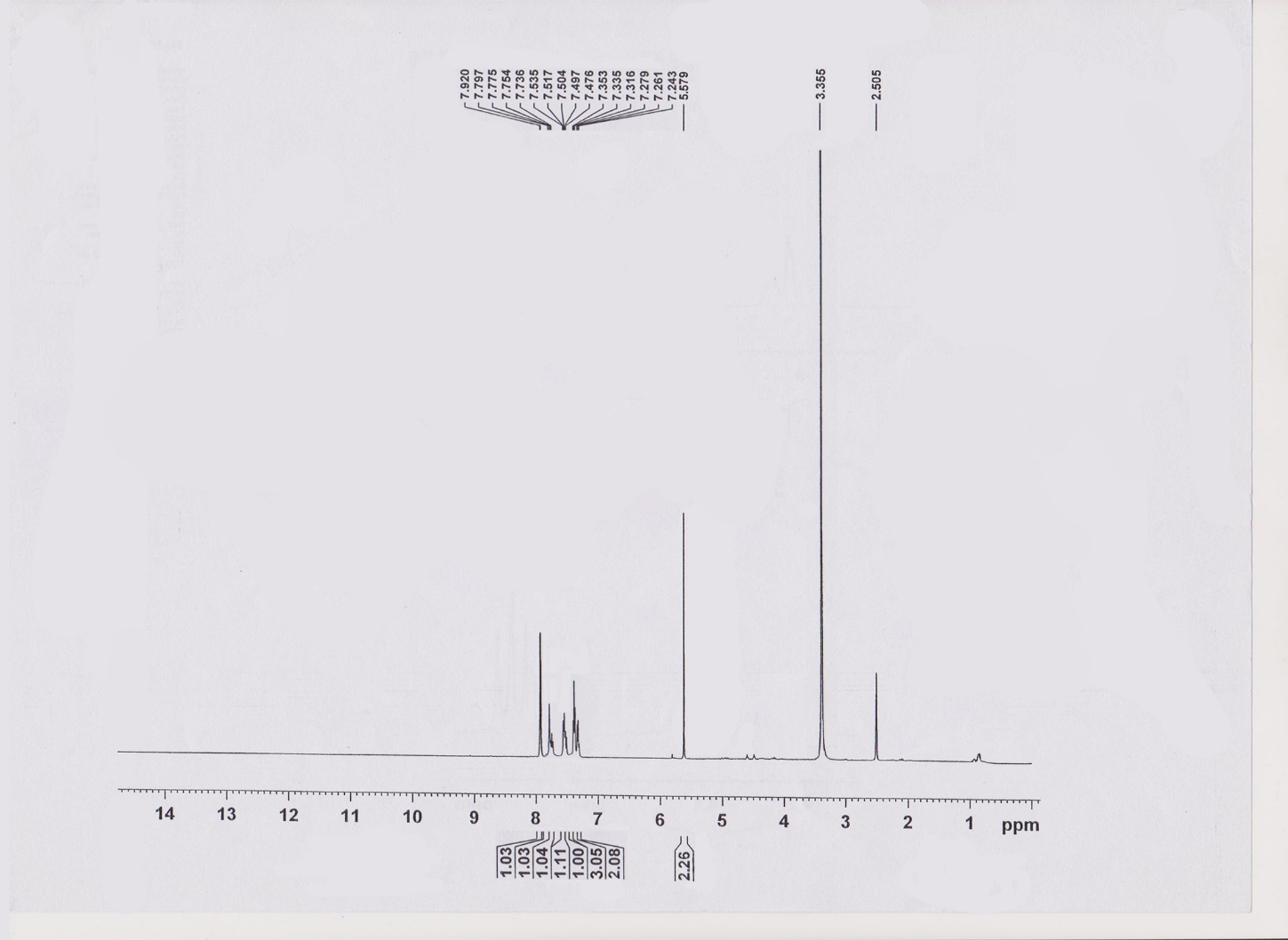


Figure 3. ^1^H NMR 4-Bromobenzyl-4-phenyl-1H-1,2,3-triazole (4b)


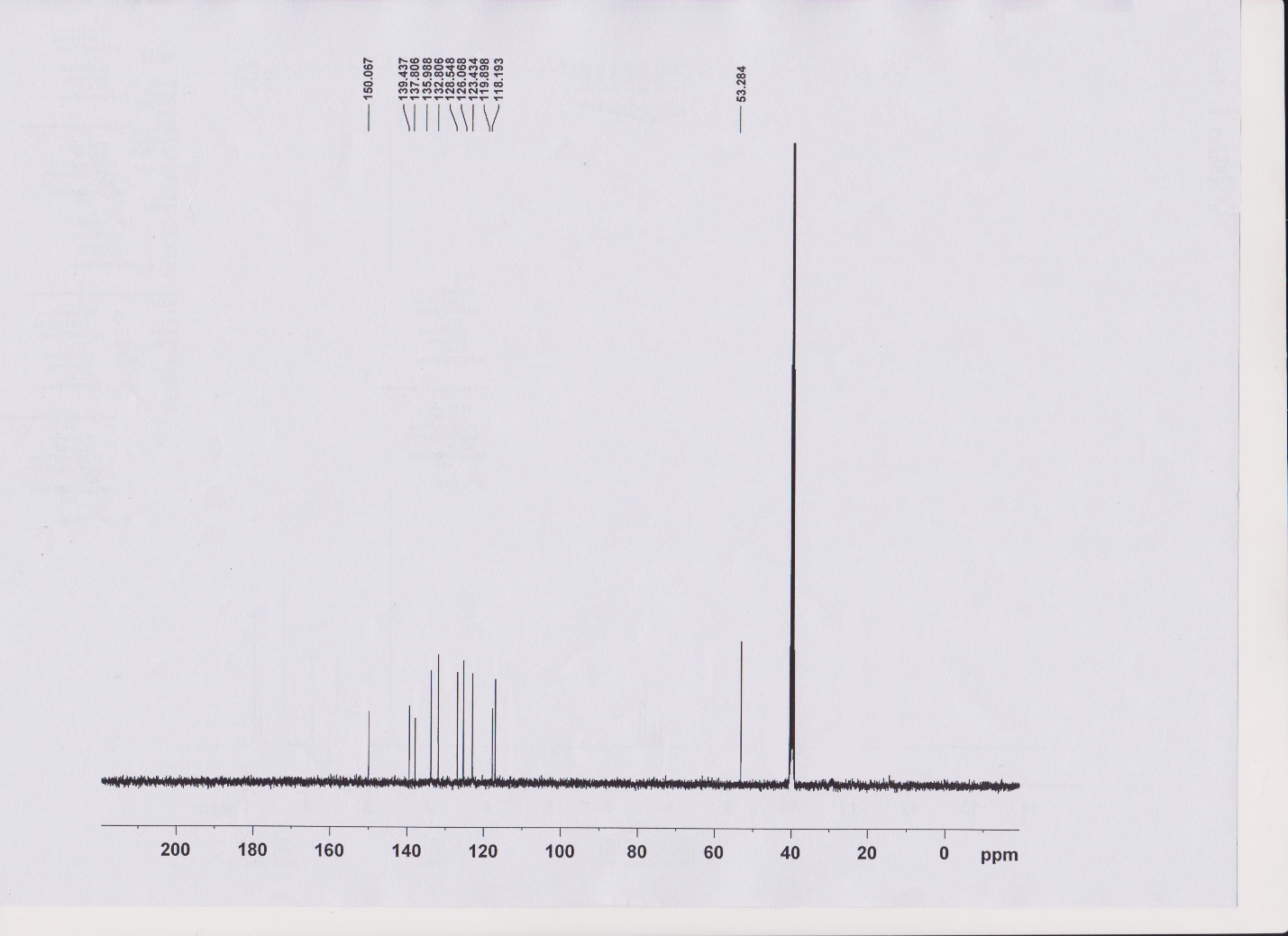


Figure 4. ^13^C NMR 4-Bromobenzyl-4-phenyl-1H-1,2,3-triazole (4b)

2.7.2. 4-Bromobenzyl-4-phenyl-1H-1,2,3-triazole (4b)

White solid, m.p. = 149–150 ºC (Lit. [1]. 151–152 ºC); IR (KBr) Ѵ (cm^-1^): 3132 (C=C–H), 2913 (–C–H), 1631 (C=C_aromatic_), 1455, 1431 (CH_2_, bending), 1349 (C–N, stretching), 695 (C–Br); ^1^H NMR (DMSO-d_6_, 400 MHz) δ (ppm): 5.57 (s, 2H, CH_2_), 7.24–7.35 (m, 5H, H_aromatic_), 7.47–7.53 (m, 2H, H_aromatic_), 7.73–7.79 (m, 2H, H_aromatic_), 7.92 (s, 1H, H_triazole_); ^13^C NMR (DMSO-d_6_, 100 MHz) δ (ppm): 53.2, 118.1, 119.8, 123.4, 126.0, 128.5, 132.8, 135.9, 137.8, 139.4, 150.0.


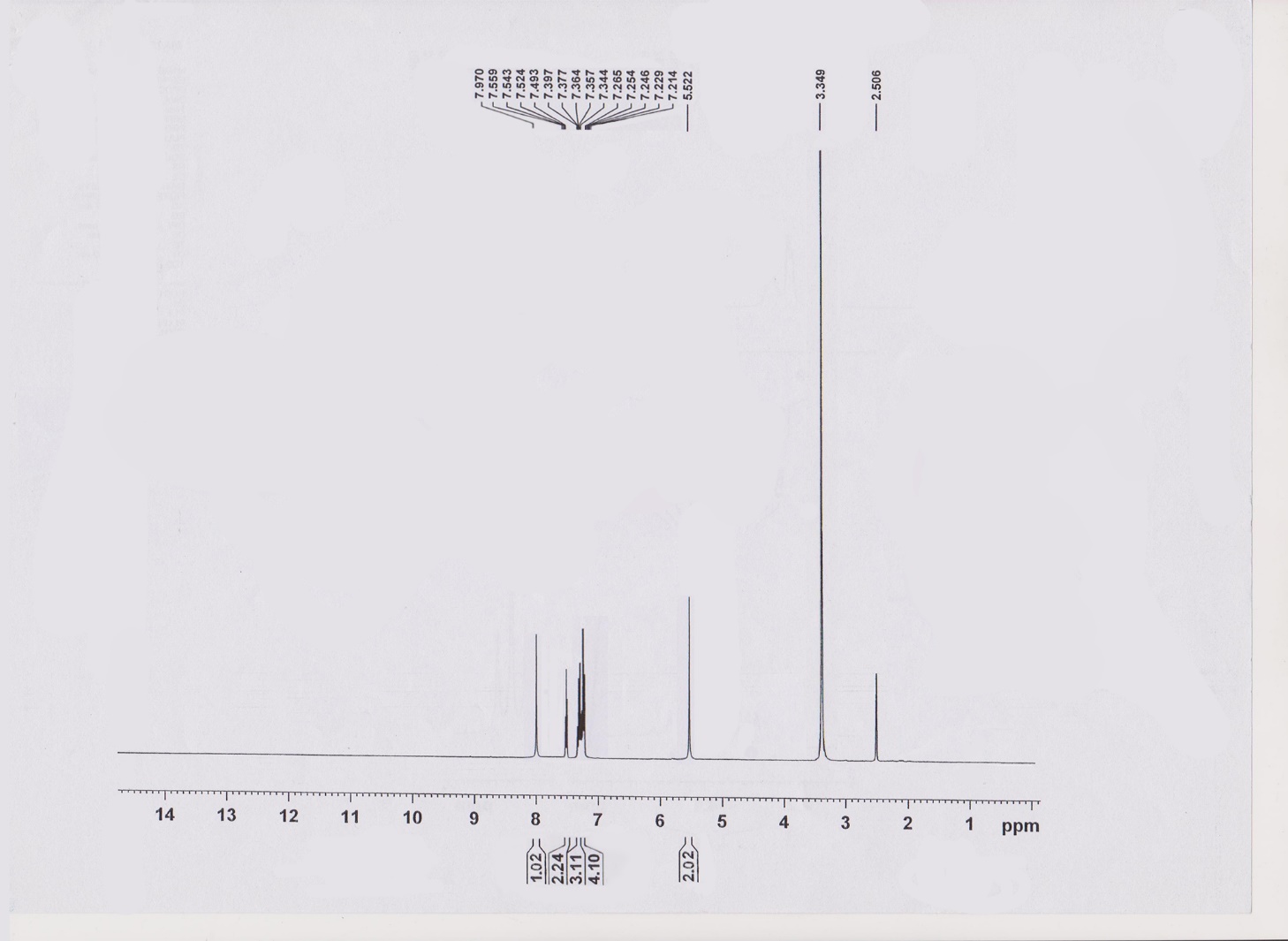


Figure 5. ^1^H NMR 1-(3-Chlorobenzyl)-4-phenyl-1H-1,2,3-triazole (4c)


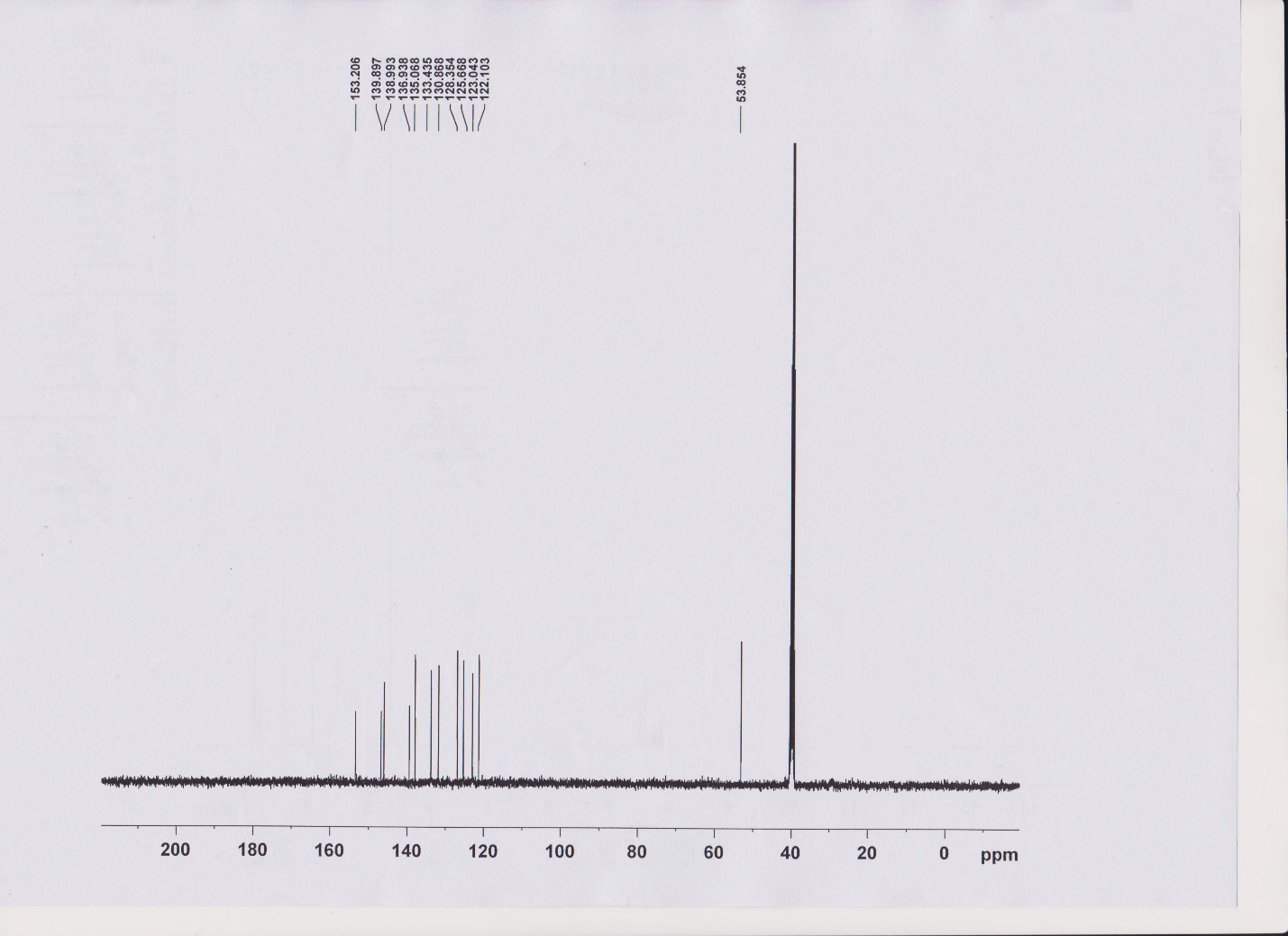


Figure 6. ^13^C NMR 1-(3-Chlorobenzyl)-4-phenyl-1H-1,2,3-triazole (4c)

2.7.3. 1-(3-Chlorobenzyl)-4-phenyl-1H-1,2,3-triazole (4c)

White solid, m.p. = 89–90 ºC (Lit. [2]. 87–89 ºC); IR (KBr) Ѵ (cm^-1^): 3077 (C=C–H), 2935 (–C–H), 1583 (C=C_aromatic_), 1454 (CH_2_, bending), 1329 (C–N, stretching), 756 (C–Cl); ^1^H NMR (DMSO-d_6_, 400 MHz) δ (ppm): 5.52 (s, 2H, CH_2_), 7.21–7.39 (m, 7H, H_aromatic_), 7.49–7.55 (m, 2H, H_aromatic_), 7.97 (s, 1H, H_triazole_); ^13^C NMR (DMSO-d_6_, 100 MHz) δ (ppm): 53.8, 122.1, 123.0, 125.6, 128.3, 130.8, 133.4, 135.0, 136.9, 138.9, 139.8, 153.2.


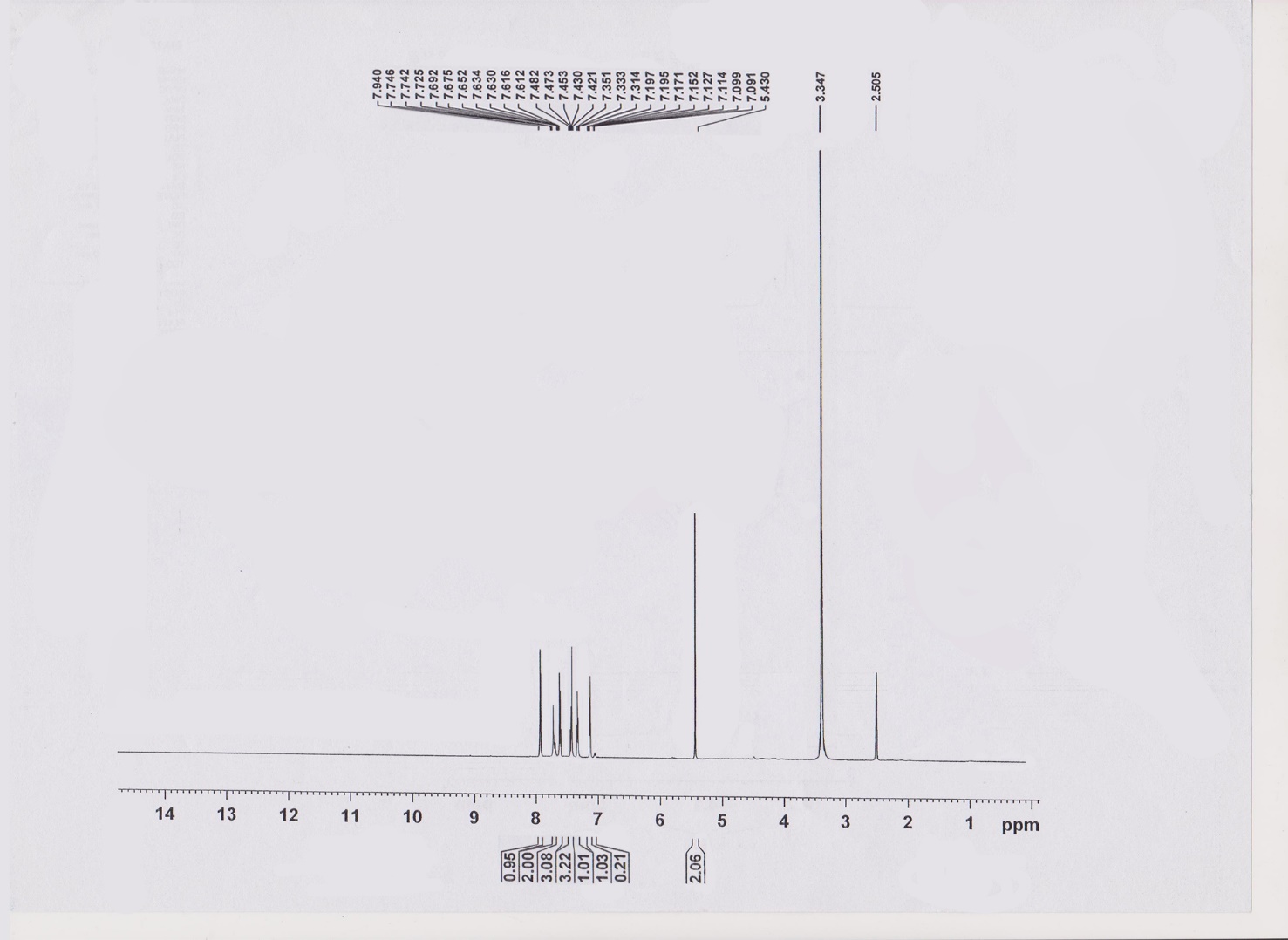


Figure 7. ^1^H NMR 1-(2-Chlorobenzyl)-4-phenyl-1H-1,2,3-triazole (4d)


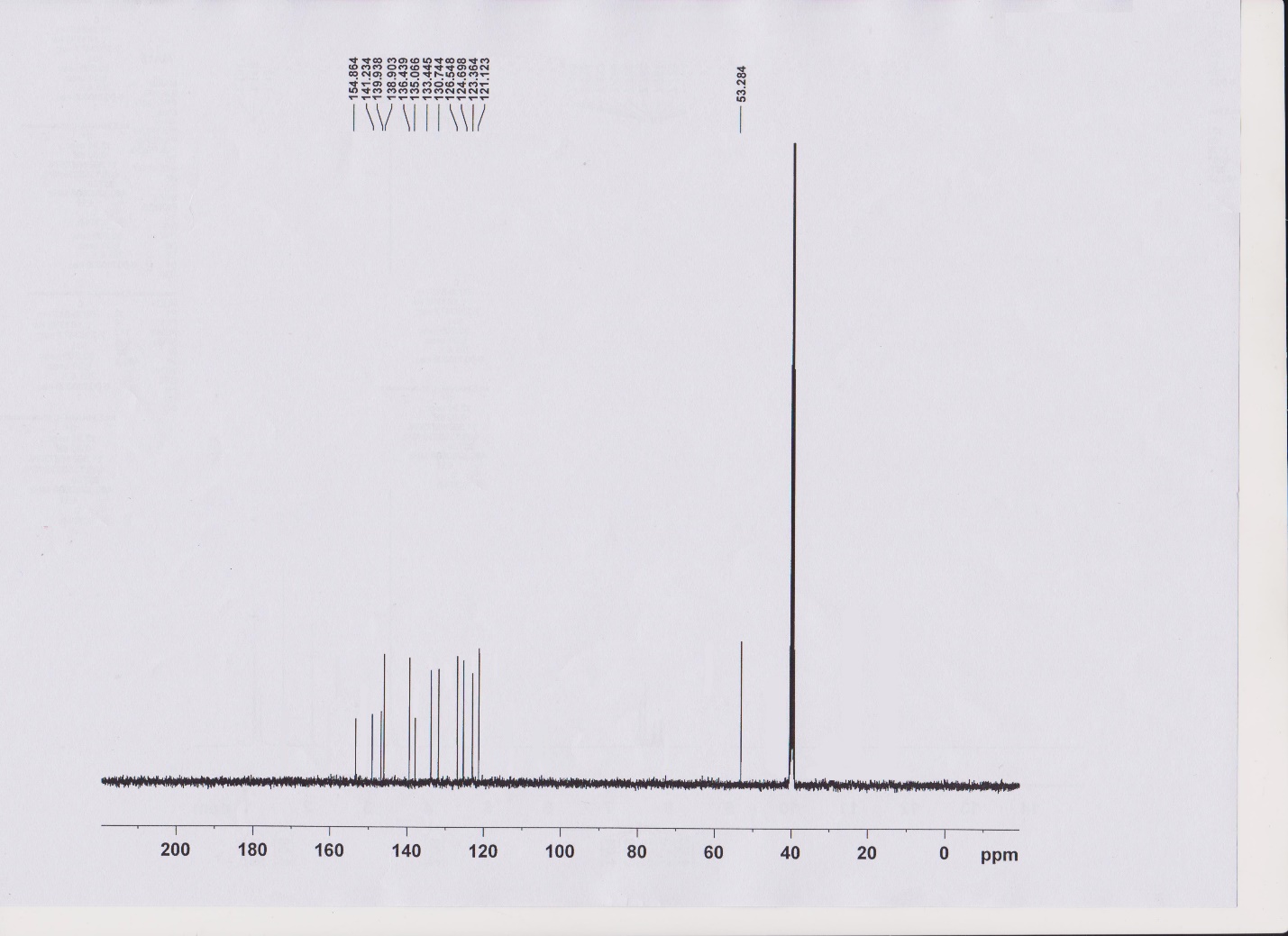


Figure 8. ^13^C NMR 1-(2-Chlorobenzyl)-4-phenyl-1H-1,2,3-triazole (4d)

2.7.4. 1-(2-Chlorobenzyl)-4-phenyl-1H-1,2,3-triazole (4d)

White solid, m.p. = 94–97 ºC (Lit. [3]. 96–97 ºC); IR (KBr) Ѵ (cm^-1^): 3142 (C=C–H), 2931 (–C–H), 1569 (C=C_aromatic_), 1459, 1422 (CH_2_, bending), 1333 (C–N, stretching), 689 (C–Cl); ^1^H NMR (DMSO-d_6_, 400 MHz) δ (ppm): 5.43 (s, 2H, CH_2_), 7.11-7.19 (m, 1H, H_aromatic_), 7.24–7.28 (t, J = 8.0 Hz, 1H, H_aromatic_), 7.42–7.48 (m, 3H, H_aromatic_), 7.61–7.67 (m, 3H, H_aromatic_), 7.69–7.74 (m, 2H, H_aromatic_), 7.94 (s, 1H, H_triazole_); ^13^C NMR (DMSO-d_6_, 100 MHz) δ (ppm): 53.2, 121.1, 123.3, 124.6, 126.5, 130.7, 133.4, 135.0, 136.4, 138.9, 139.9, 141.2, 154.8.


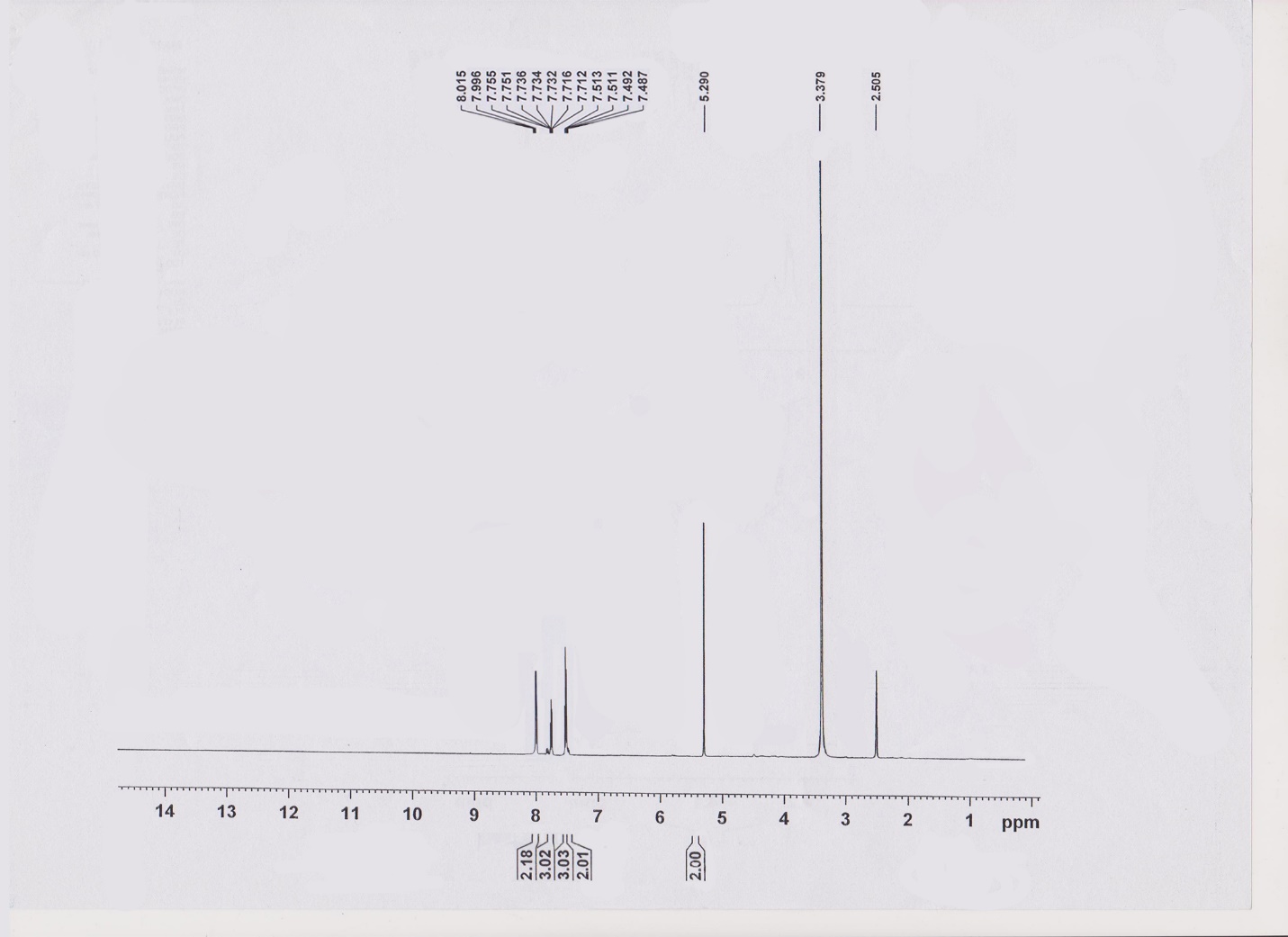


Figure 9. ^1^H NMR 1-(4-Nitrobenzyl)-4-phenyl-1H-1,2,3-triazole (4e)


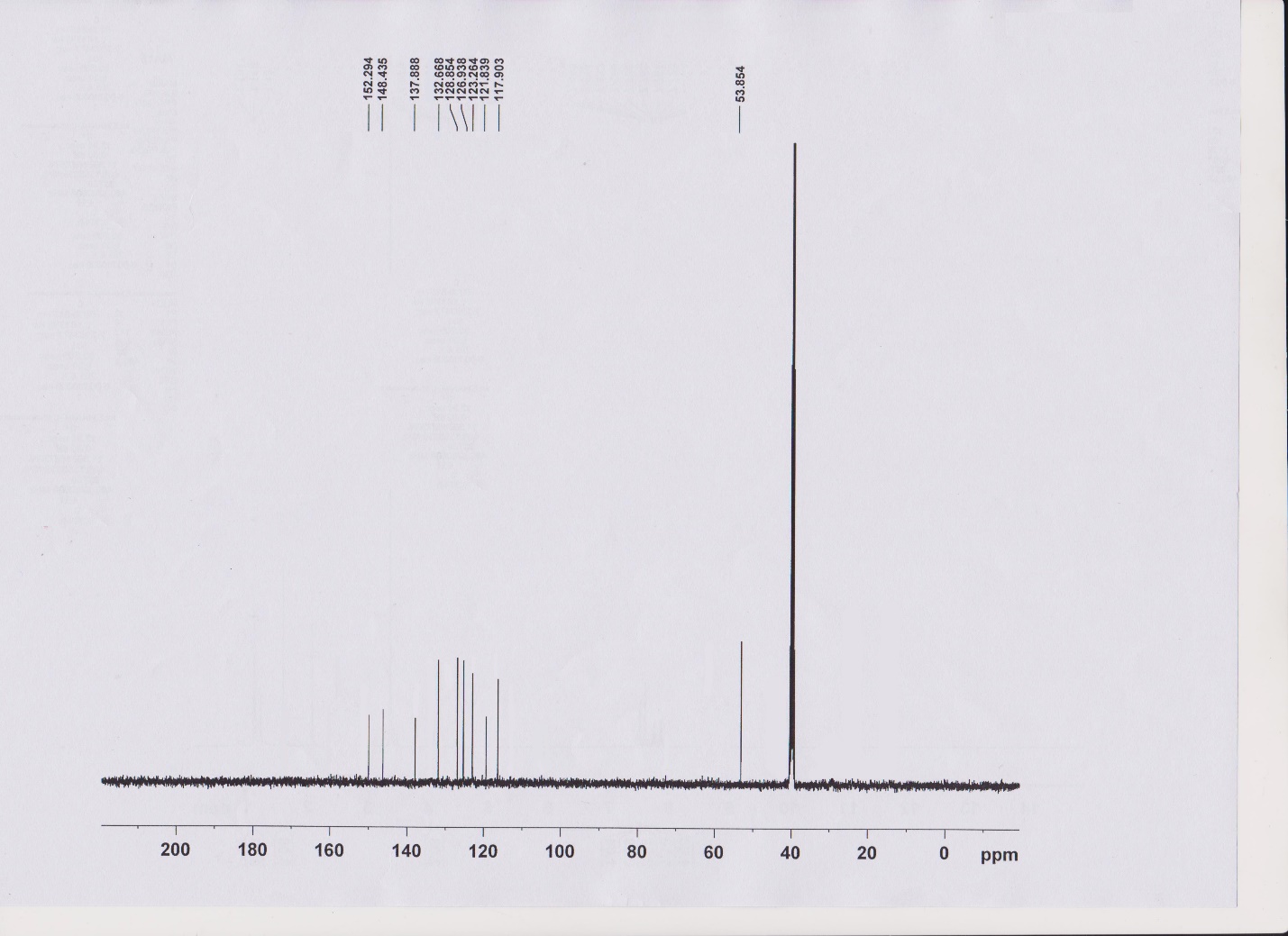


Figure 10. ^13^C NMR 1-(4-Nitrobenzyl)-4-phenyl-1H-1,2,3-triazole (4e)

2.7.5. 1-(4-Nitrobenzyl)-4-phenyl-1H-1,2,3-triazole (4e)

Pale yellow solid, m.p. = 142–143 ºC (Lit. [1]. 140–141 ºC); IR (KBr) Ѵ (cm^-1^): 3099 (C=C–H), 1587 (C=C), 1353, 1522 (–NO_2_), 1465 (–CH_2_, bending), 1256 (C–N); ^1^H NMR (DMSO-d_6_, 400 MHz) δ (ppm): 5.29 (s, 2H, CH_2_), 7.48–7.51 (m, 5H, H_aromatic_), 7.71–7.75 (m, 3H, H_aromatic_, H_triazole_), 8.31 (d, J = 7.6 Hz, 2H, H_aromatic_); ^13^C NMR (DMSO-d_6_, 100 MHz) δ (ppm): 53.8, 117.9, 121.8, 123.2, 126.9, 128.8, 132.6, 137.8, 148.4, 152.2.


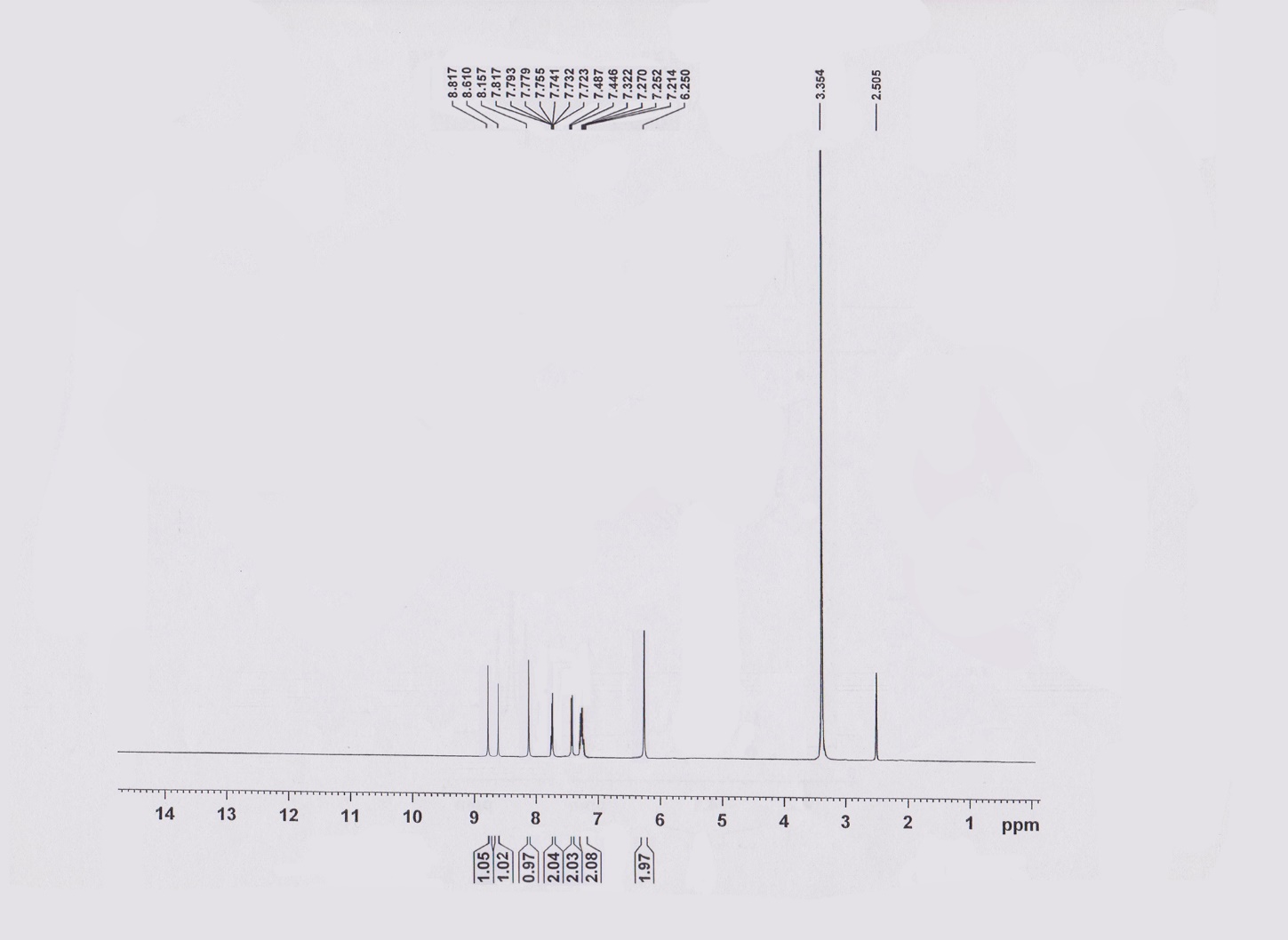


Figure 11. ^1^H NMR 1-(2,4-Dinitrobenzyl)-4-phenyl-1H-1,2,3-triazole (4f)


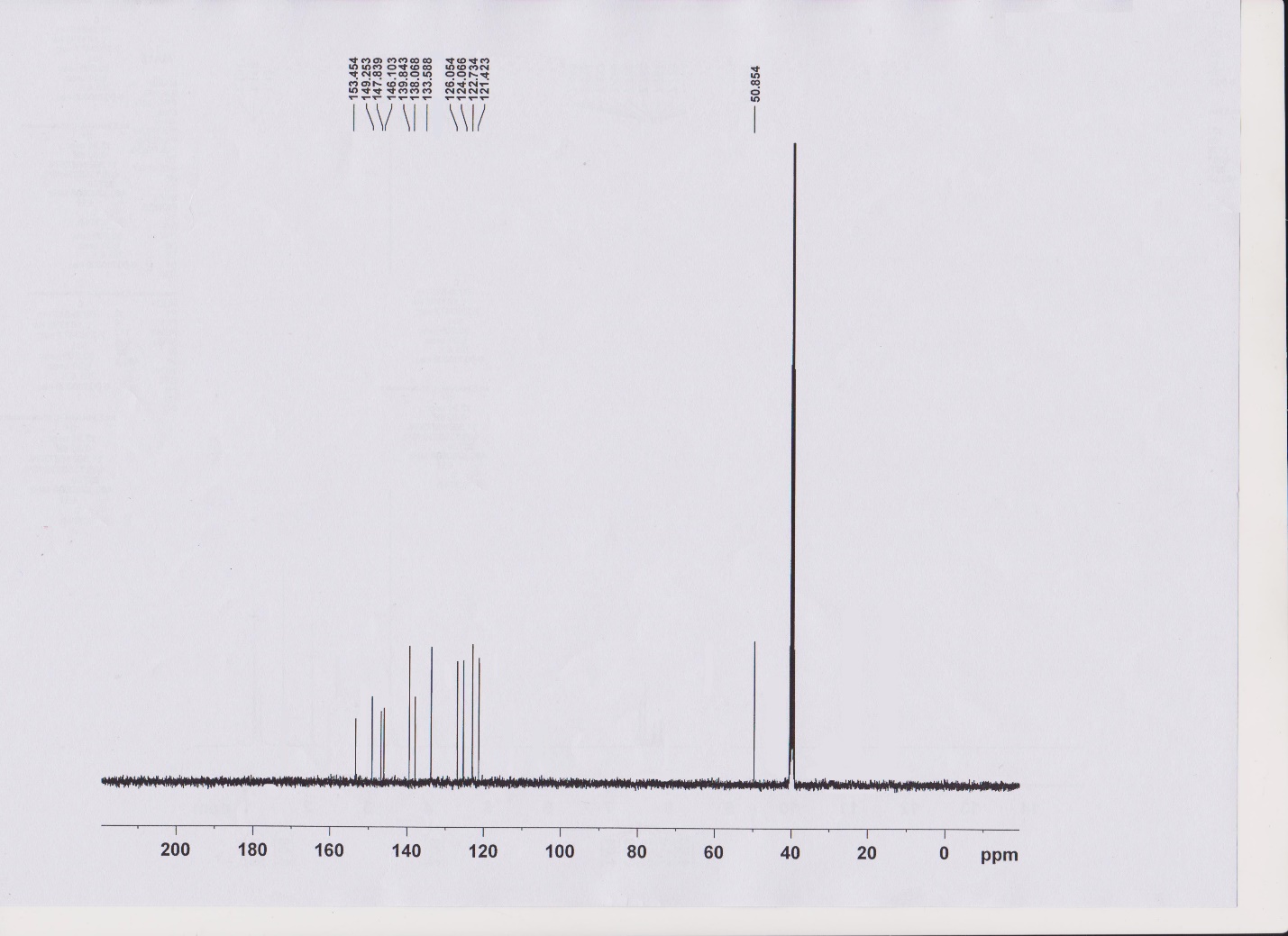


Figure 12. ^13^C NMR 1-(2,4-Dinitrobenzyl)-4-phenyl-1H-1,2,3-triazole (4f)

2.7.6. 1-(2,4-Dinitrobenzyl)-4-phenyl-1H-1,2,3-triazole (4f)

Pale yellow solid, m.p. = 212–214 ºC (Lit. [4]. 214–215 ºC); IR (KBr) Ѵ (cm^-1^): 3132 (C=C–H), 1645 (C=C_aromatic_), 1548, 1326 (–NO_2_), 1487 (CH_2_, stretching); ^1^H NMR (DMSO-d_6_, 400 MHz) δ (ppm): 6.25 (s, 2H, CH_2_), 7.21–7.32 (m, 2H, H_aromatic_), 7.44–7.48 (d, J = 8 Hz, 2H, H_aromatic_), 7.72–7.81 (m, 2H, H_aromatic_), 8.15 (d, 1H, H_triazole_), 8.61 (s, 1H, H_aromatic_), 8.81 (s, 1H, H_aromatic_); ^13^C NMR (DMSO-d_6_, 100 MHz) δ (ppm): 50.8, 121.4, 122.7, 124.0, 126.0, 133.5, 138.0, 139.8, 146.1, 147.8, 149.2, 153.4.


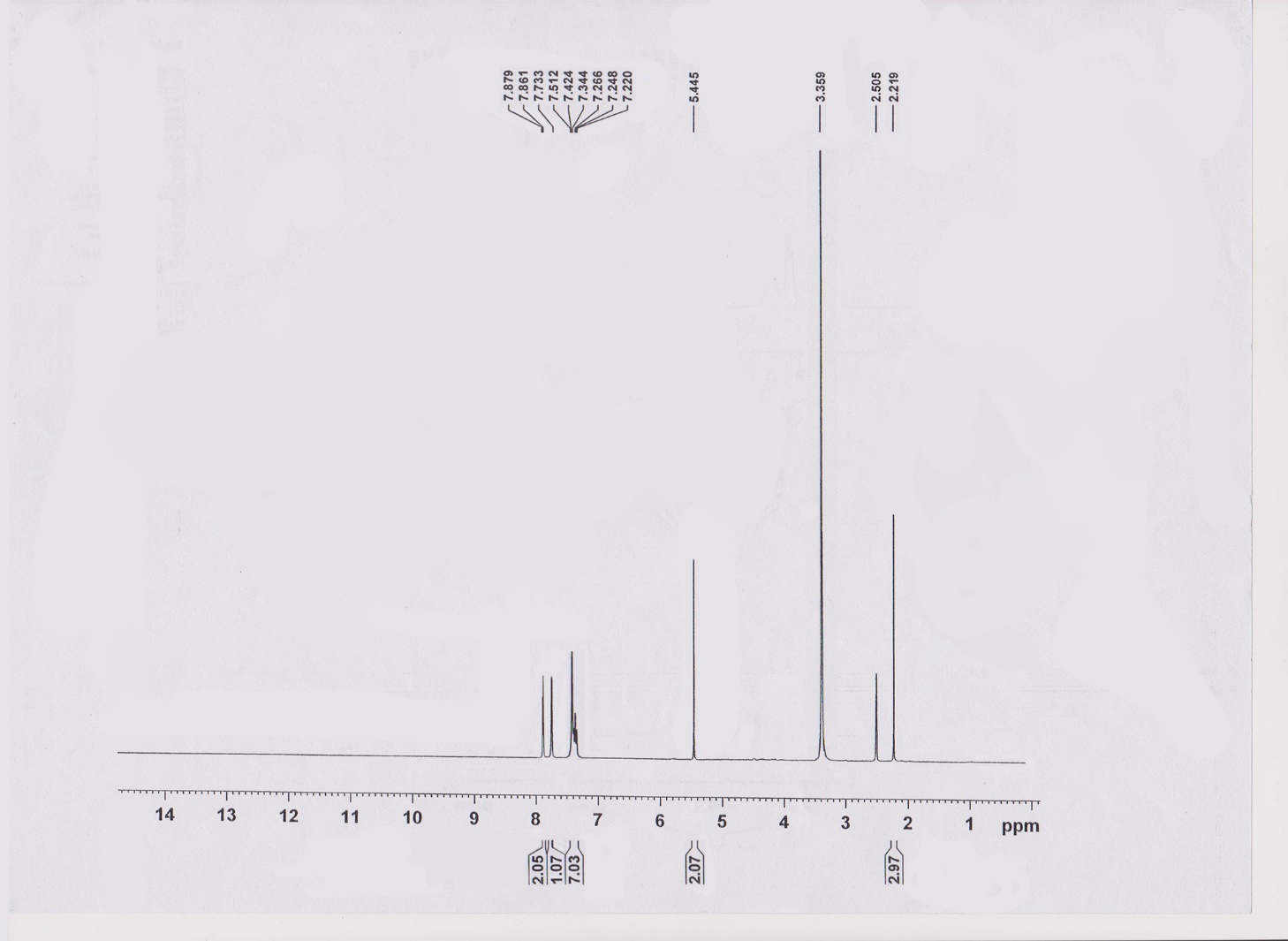


Figure 13. ^1^H NMR 1-Benzyl-4-(4-methylphenyl)-1H-1,2,3-triazol (4g)


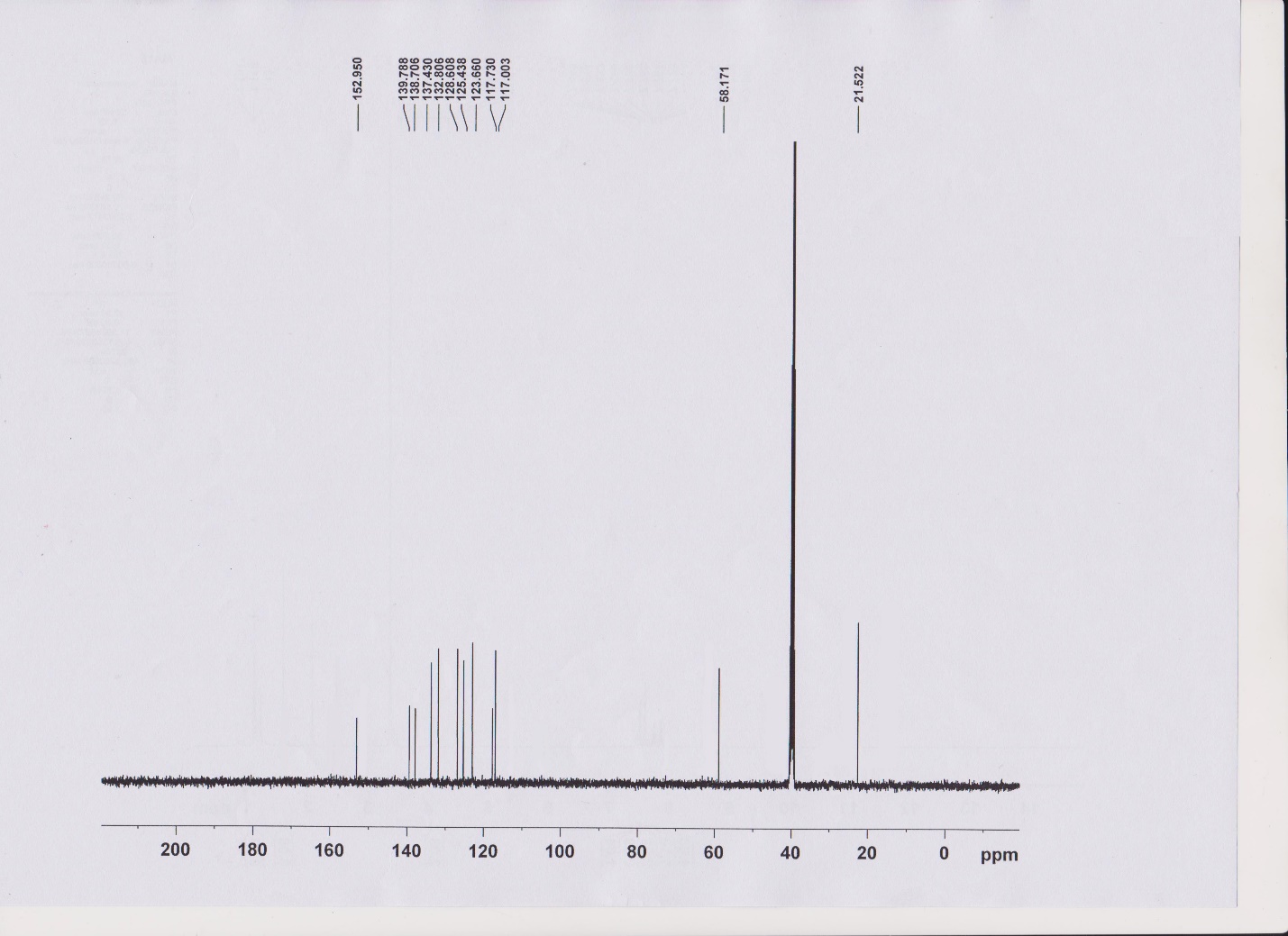


Figure 14. ^13^C NMR 1-Benzyl-4-(4-methylphenyl)-1H-1,2,3-triazol (4g)

2.7.7. 1-Benzyl-4-(4-methylphenyl)-1H-1,2,3-triazol (4g)

Pale yellow solid, m.p. = 150–153 ºC (Lit. [5]. 152–154 ºC); IR (KBr) Ѵ (cm^-1^): 3111 (C=C–H), 2944 (–C–H), 1575 (C=C_aromatic_), 1324 (C–N, stretching); ^1^H NMR (DMSO-d_6_, 400 MHz) δ (ppm): 2.21 (s, 3H, CH_3_), 5.44 (s, 2H, CH_2_), 7.22–7.51 (m, 7H, H_aromatic_), 7.73 (s, 1H, H_triazole_), 7.87 (d, J = 7.6 Hz, 2H, H_aromatic_); ^13^C NMR (DMSO-d_6_, 100 MHz) δ (ppm): 21.5, 58.1, 117.0, 117.7, 123.6, 125.4, 128.6, 132.8, 137.4, 138.7, 139.7, 152.9.


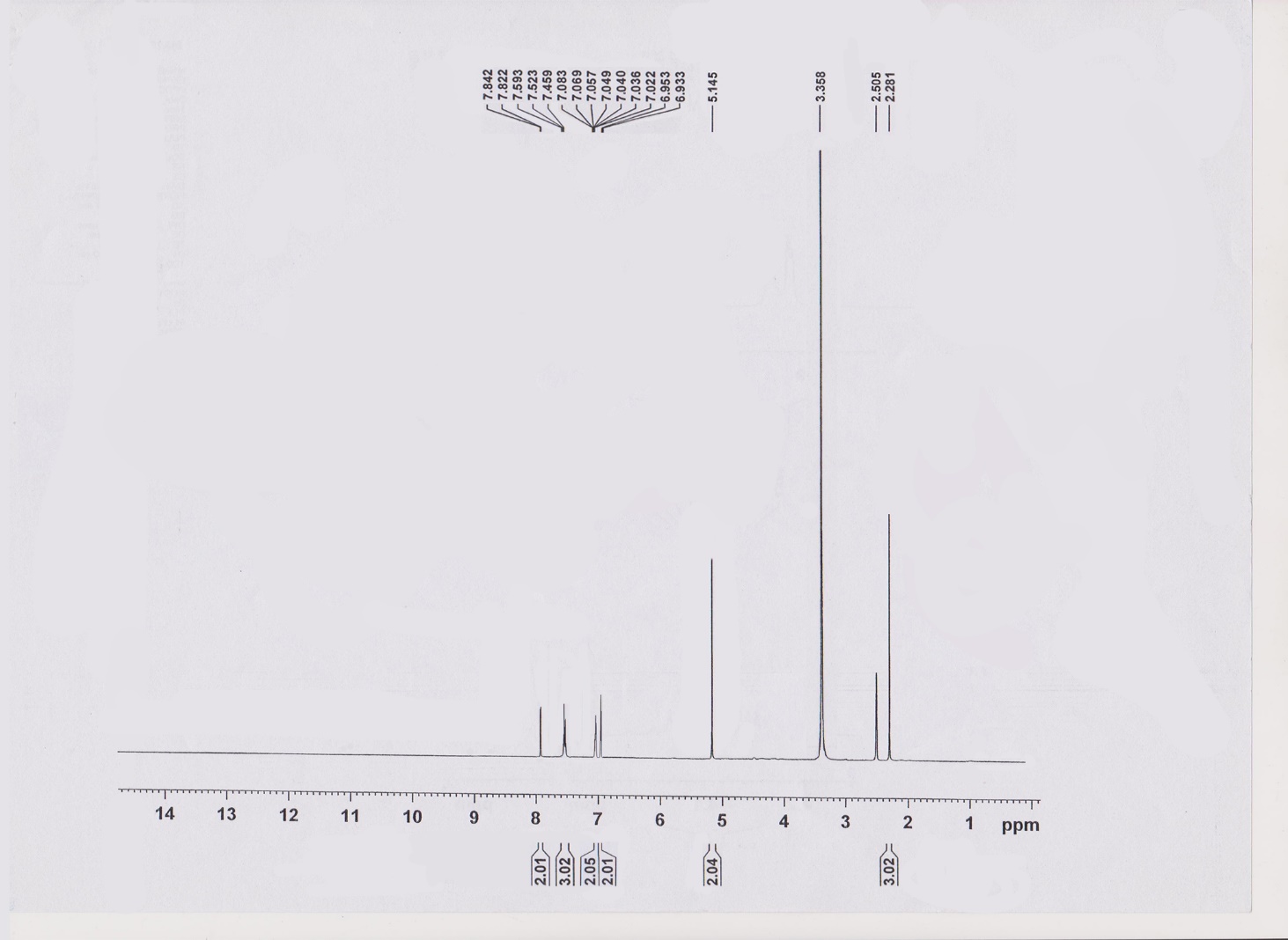


Figure 15. ^1^H NMR 1-(4-Bromobenzyl)-4-(4-methylphenyl)-1H-1,2,3-triazole (4h)


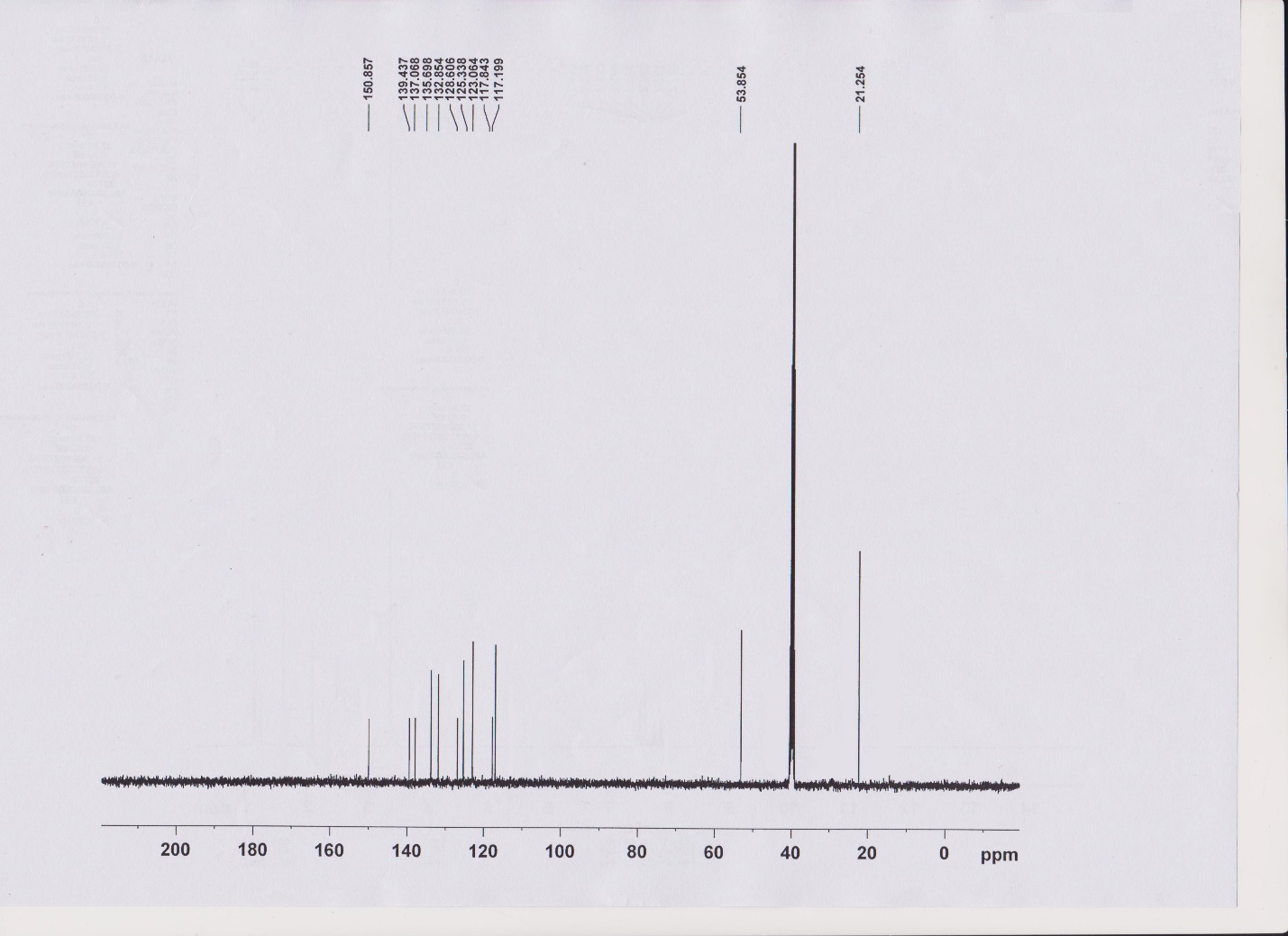


Figure 16. ^13^C NMR 1-(4-Bromobenzyl)-4-(4-methylphenyl)-1H-1,2,3-triazole (4h)

2.7.8. 1-(4-Bromobenzyl)-4-(4-methylphenyl)-1H-1,2,3-triazole (4h)

Pale yellow solid, m.p. = 201–203 ºC (Lit. [4]. 202–204 ºC); IR (KBr) Ѵ (cm^-1^): 3099 (C=C–H), 1611 (C=C_aromatic_), 1335 (C–N, stretching), 714 (C–Br); ^1^H NMR (DMSO-d_6_, 400 MHz) δ (ppm): 2.28 (s, 3H, CH_3_), 5.14 (s, 2H, CH_2_), 6.94 (d, J = 8.0 Hz, 2H, H_aromatic_), 7.02–7.08 (m, 2H, H_aromatic_), 7.45–7.59 (m, 3H, H_aromatic_, H_triazole_), 7.83 (d, J = 8.0 Hz, 2H, H_aromatic_); ^13^C NMR (DMSO-d_6_, 100 MHz) δ (ppm): 21.2, 53.8, 117.1, 117.8, 123.0, 125.3, 128.6, 132.8, 135.6, 137.0, 139.4, 150.8.


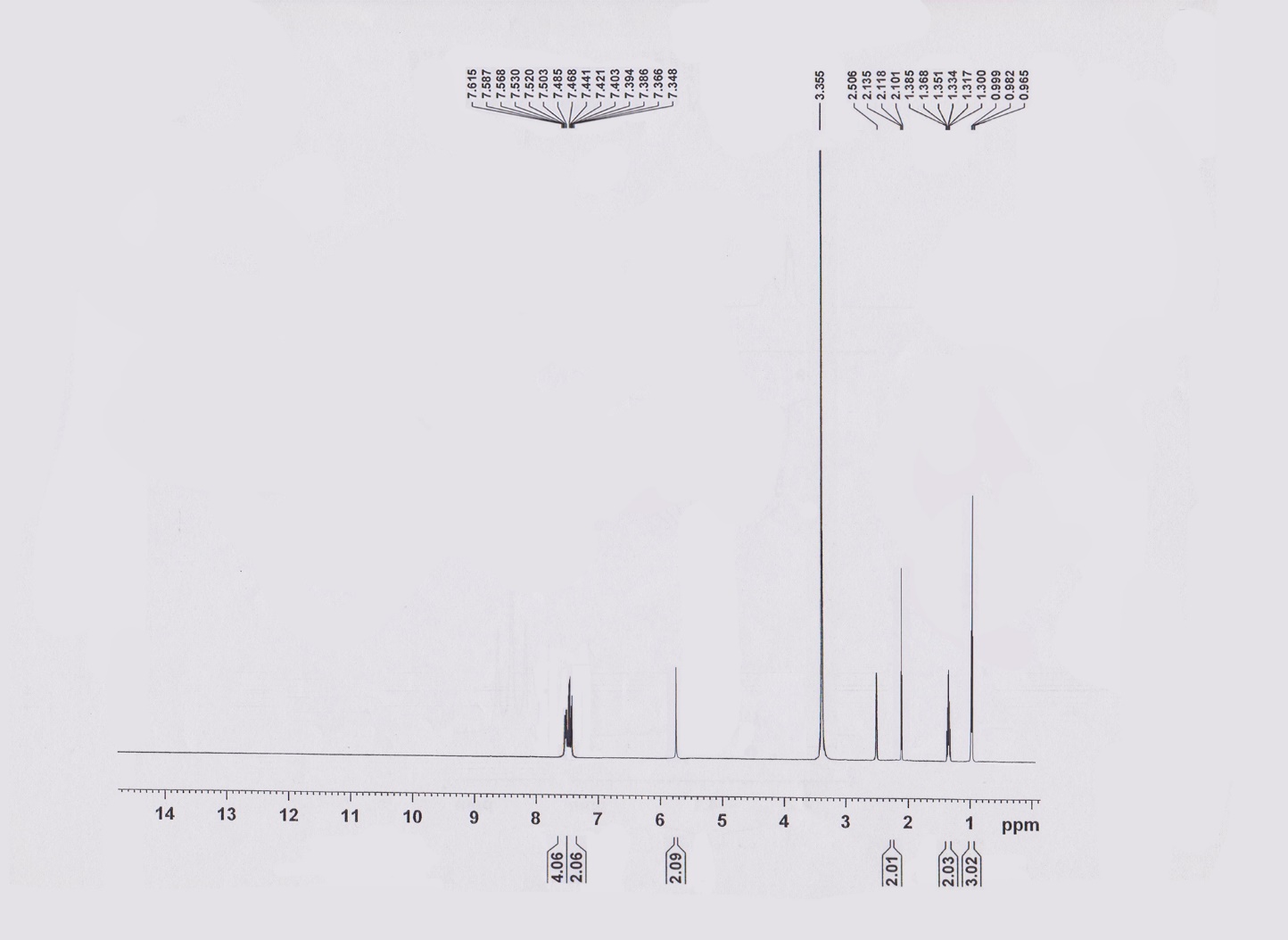


Figure 17. ^1^H NMR 1-Benzyl-4-propyl-1H-1,2,3-triazole (4i)


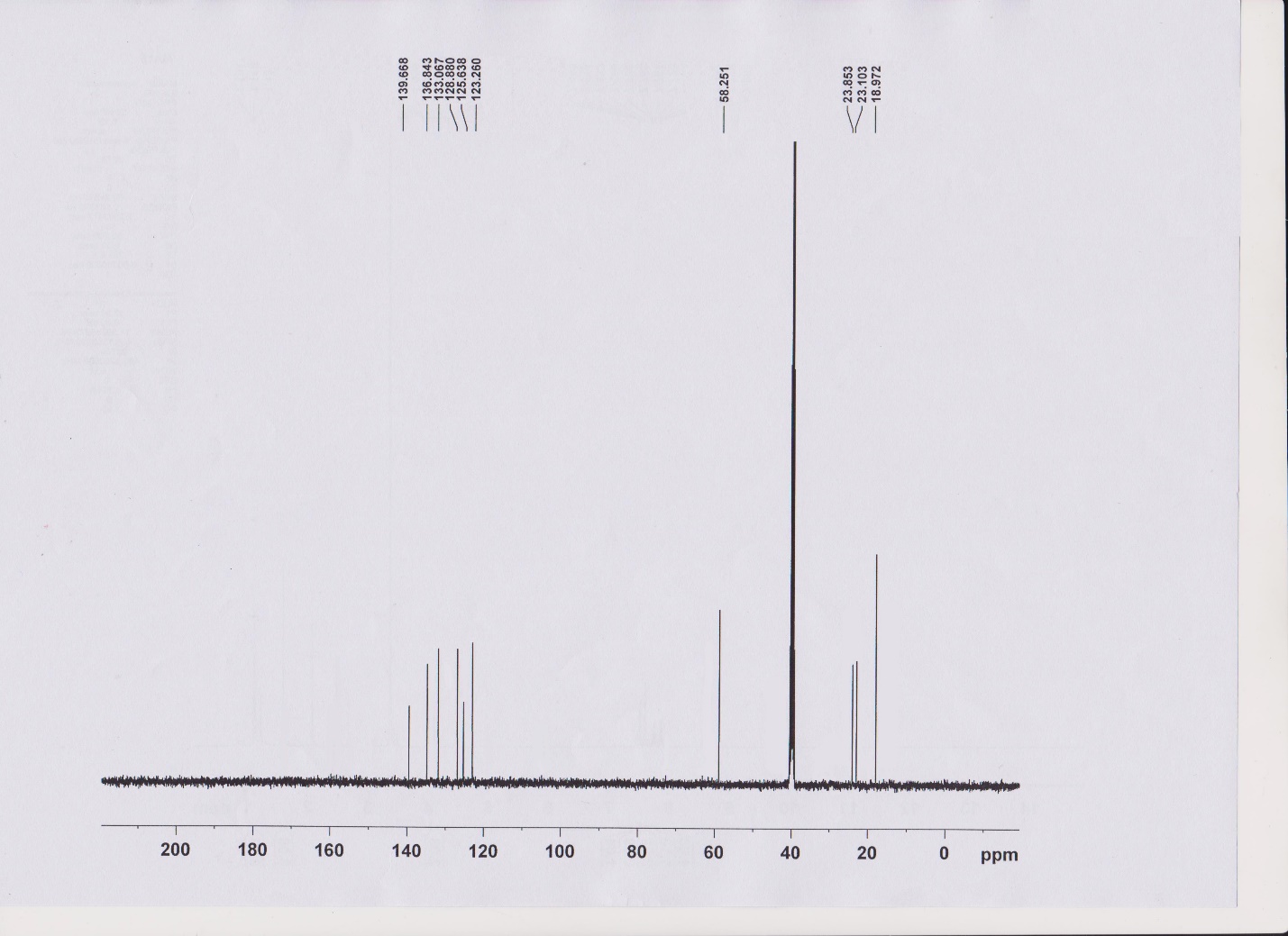


Figure 18. ^13^C NMR 1-Benzyl-4-propyl-1H-1,2,3-triazole (4i)

2.7.9. 1-Benzyl-4-propyl-1H-1,2,3-triazole (4i)

Greenish yellow oil (Lit. [3, 6]); IR (KBr) Ѵ (cm^-1^): 2939 (C=C–H), 1586 (C=C_aromatic_), 1221 (C–N, stretching); ^1^H NMR (DMSO-d_6_, 400 MHz) δ (ppm): 0.98 (t, J = 6.8 Hz, 3H, CH_3_), 1.34 (s, J = 6.8 Hz, 2H, –CH_2_), 2.11 (t, J = 6.8 Hz, 2H, –CH_2_), 5.74 (s, 2H, –CH_2_), 7.34–7.61 (m, 6H, H_aromatic_, H_triazole_); ^13^C NMR (DMSO-d_6_, 100 MHz) δ (ppm): 18.9, 23.1, 23.8, 58.2, 123.2, 125.6, 128.8, 133.0, 136.8, 139.6.

**Ullmann Reaction:**

**
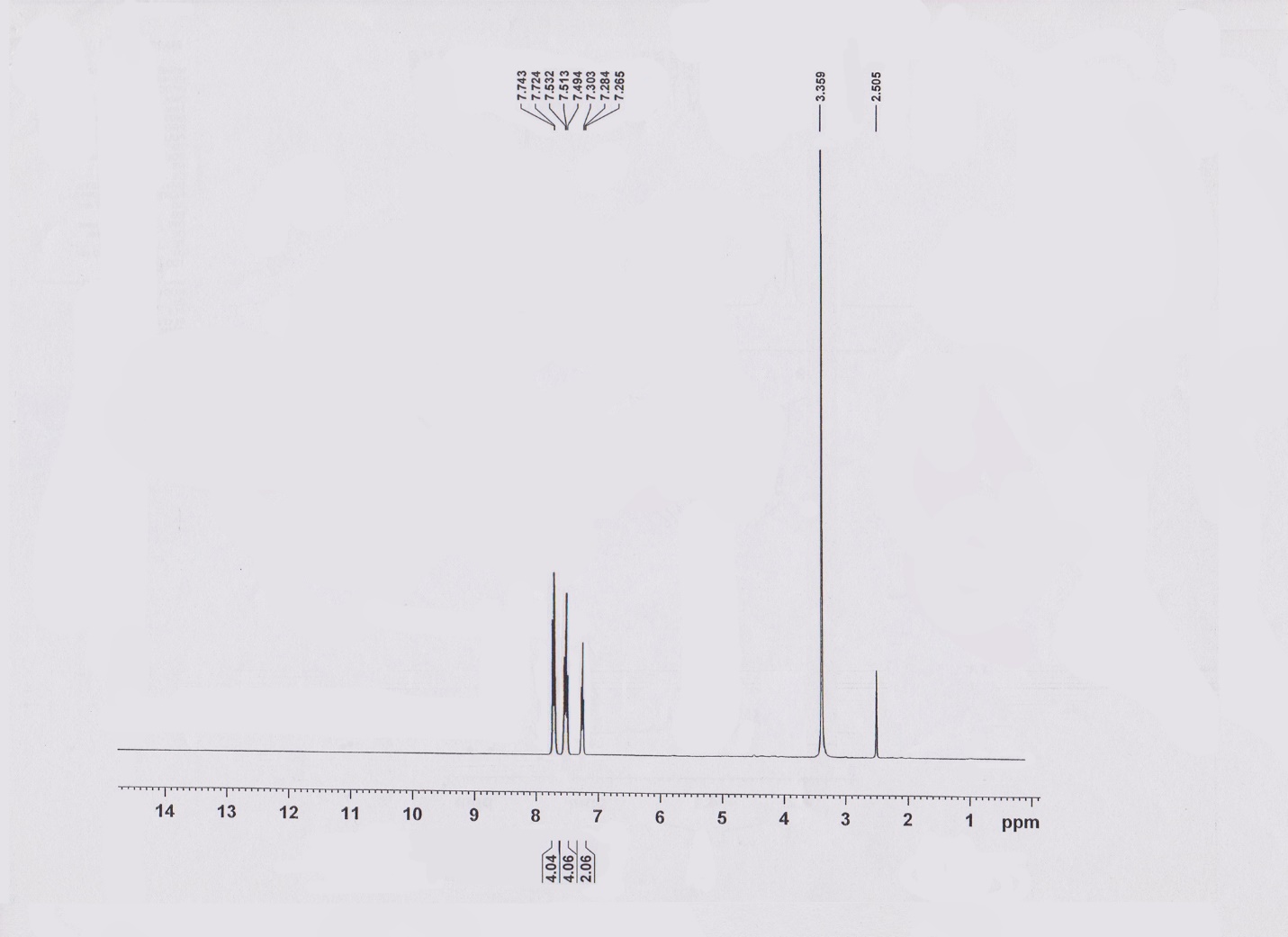
**

Figure 19. ^1^H NMR Biphenyl (6a)

**
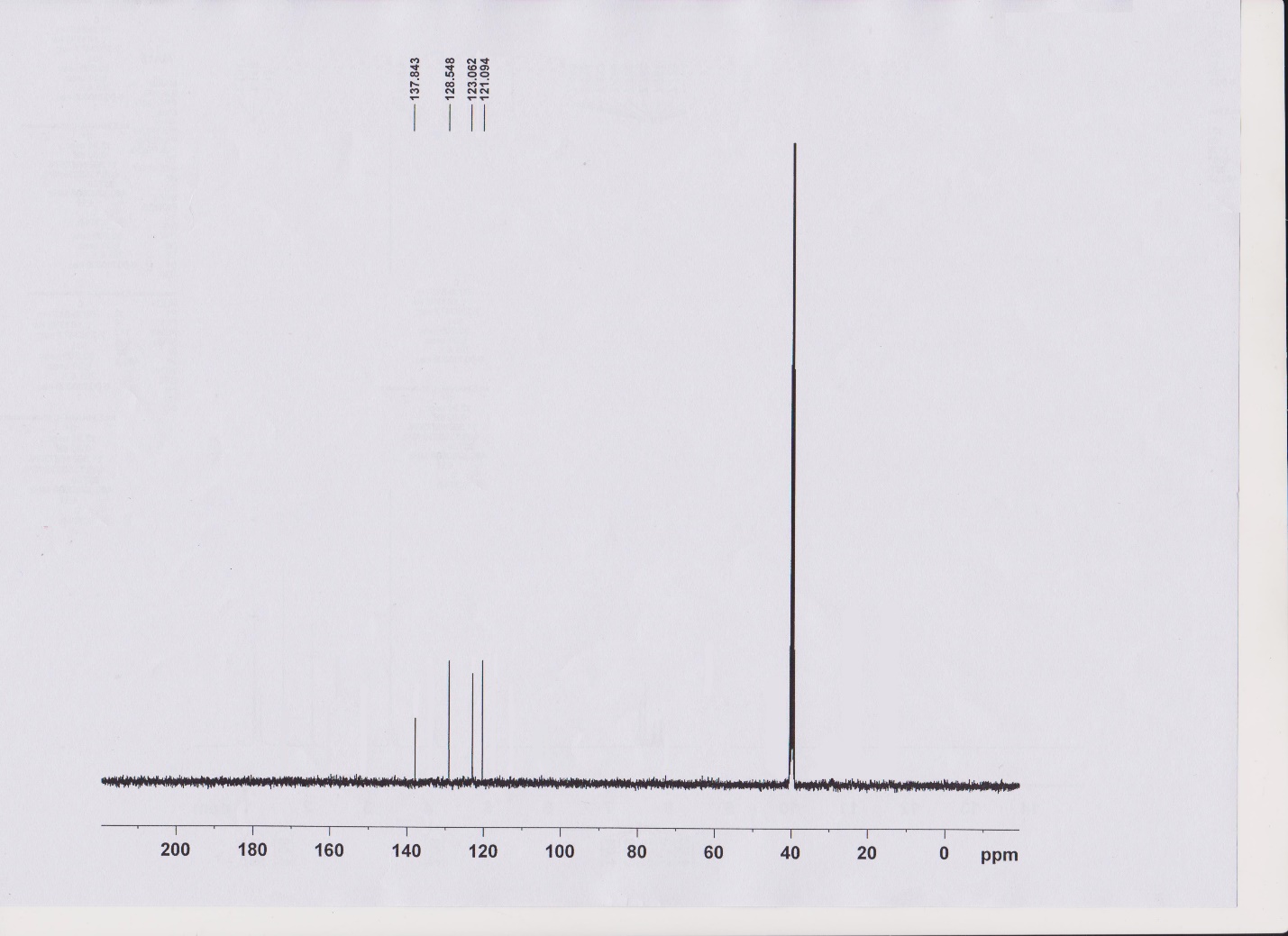
**

Figure 20. ^13^C NMR Biphenyl (6a)

2.8.1. Biphenyl (6a)

White solid, m.p. 66–68 °C (Lit. [7]. 68–69 ºC); IR (KBr) Ѵ (cm^-1^): 3052 (C=C–H, stretch), 1499 (C=C_aromatic_), 719 and 684 (C=C–H, bending); ^1^H NMR (DMSO-d_6_, 400 MHz) δ (ppm): 7.28 (t, J = 7.6 Hz, 2H, H_aromatic_), 7.51 (t, J = 7.6 Hz, 4H, H_aromatic_), 7.73 (d, J = 7.6 Hz, 4H, H_aromatic_); ^13^C NMR (DMSO-d_6_, 100 MHz) δ (ppm): 121.0, 123.0, 128.5, 137.8.


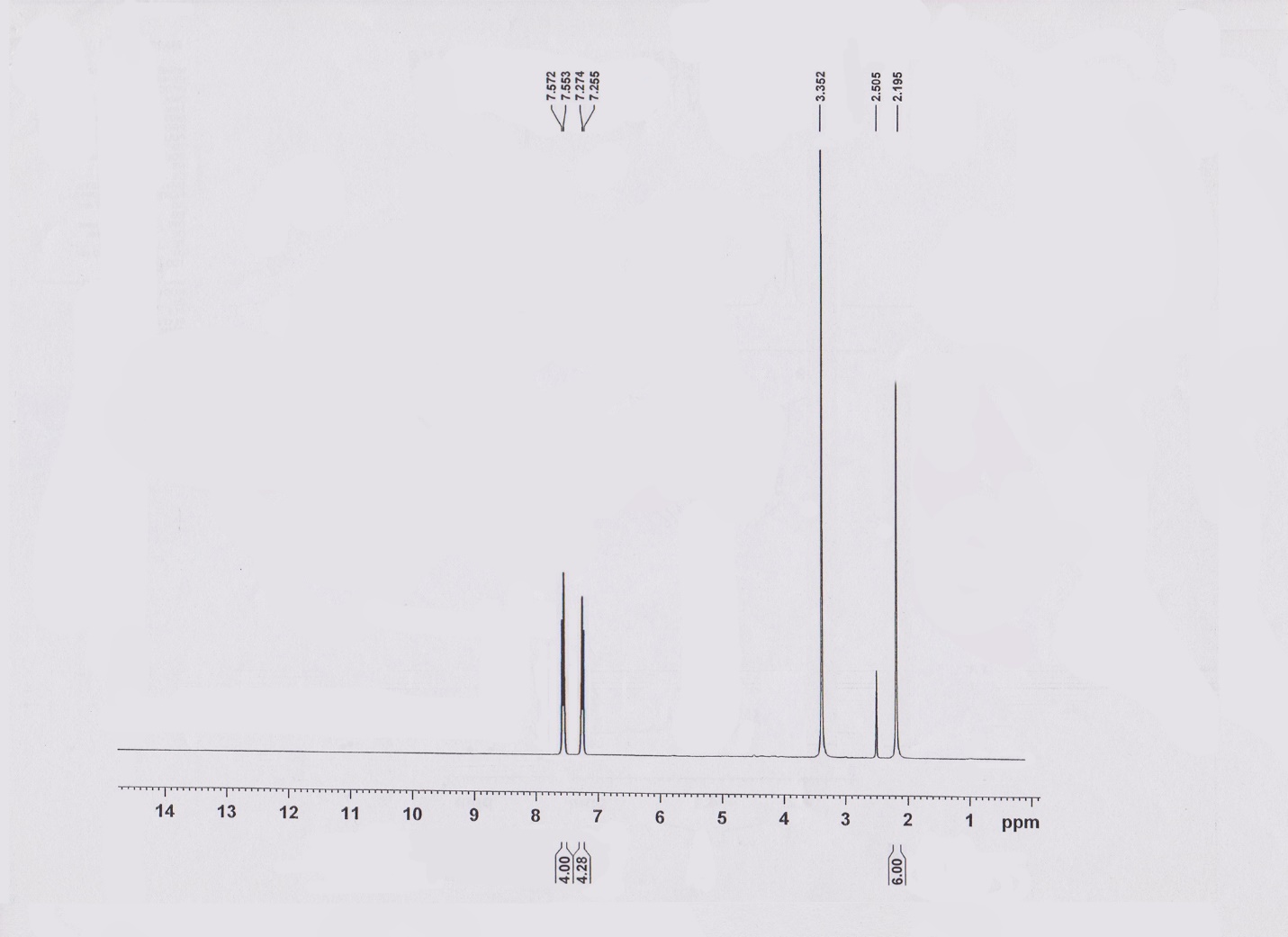


Figure 21. ^1^H NMR 4,4’-Dimethylbiphenyl (6b)

**
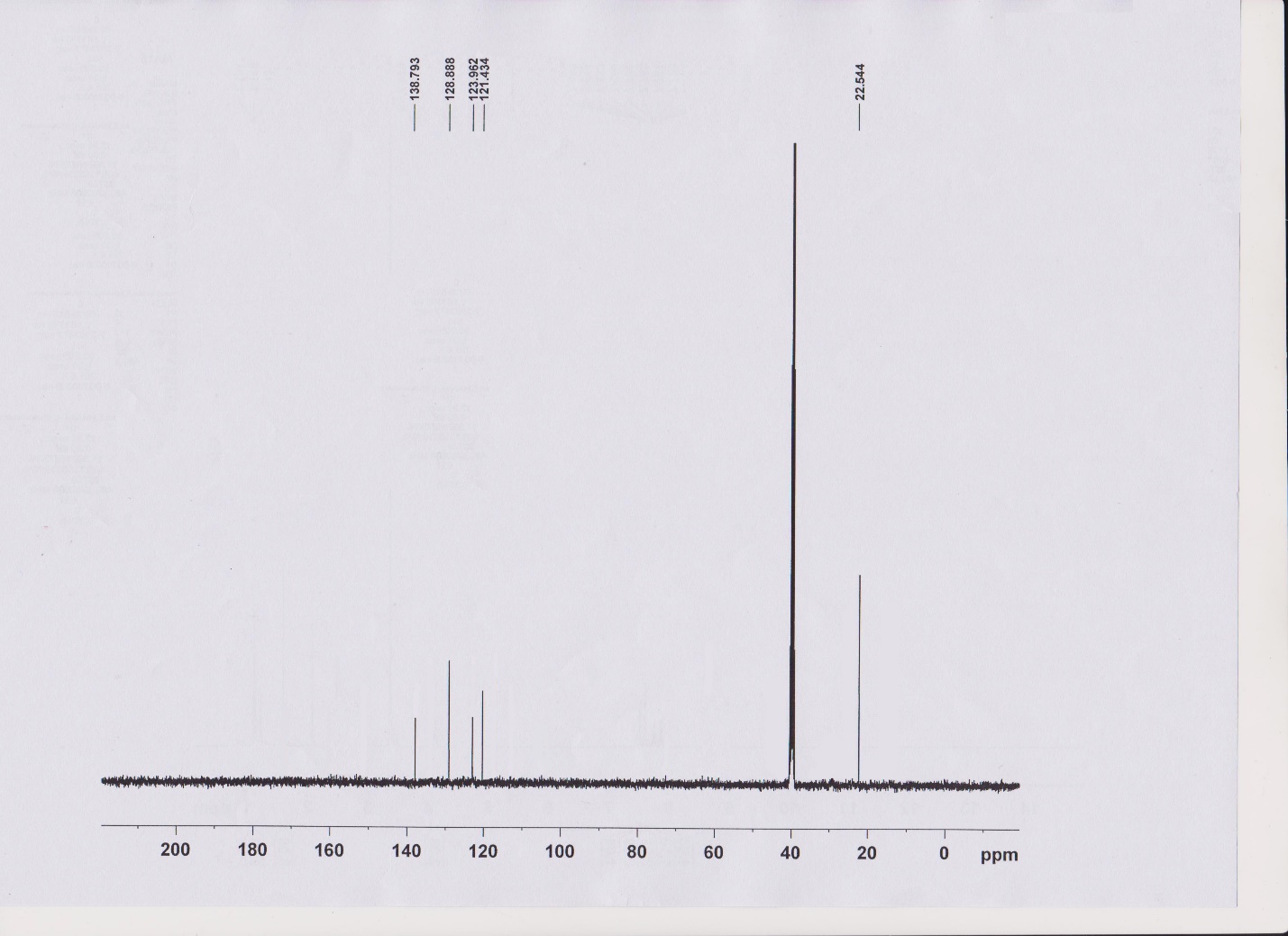
**

Figure 22. ^13^C NMR 4,4’-Dimethylbiphenyl (6b)

2.8.2. 4,4’-Dimethylbiphenyl (6b)

White solid m.p. 120–122 °C (Lit. [7]. 119–120 ºC); IR (KBr) Ѵ (cm^-1^): 3044 (C=C–H, stretch); 2926 (–C–H), 1512 (C=C_aromatic_), 810 (C=C–H, bending); ^1^H NMR (DMSO-d_6_, 400 MHz) δ (ppm): 2.19 (s, 6H), 7.26 (d, J = 7.6 Hz, 4H, H_aromatic_), 7.56 (d, J = 7.6 Hz, 4H, H_aromatic_); ^13^C NMR (DMSO-d_6_, 100 MHz) δ (ppm): 22.5, 121.4, 123.9, 128.8, 138.7.


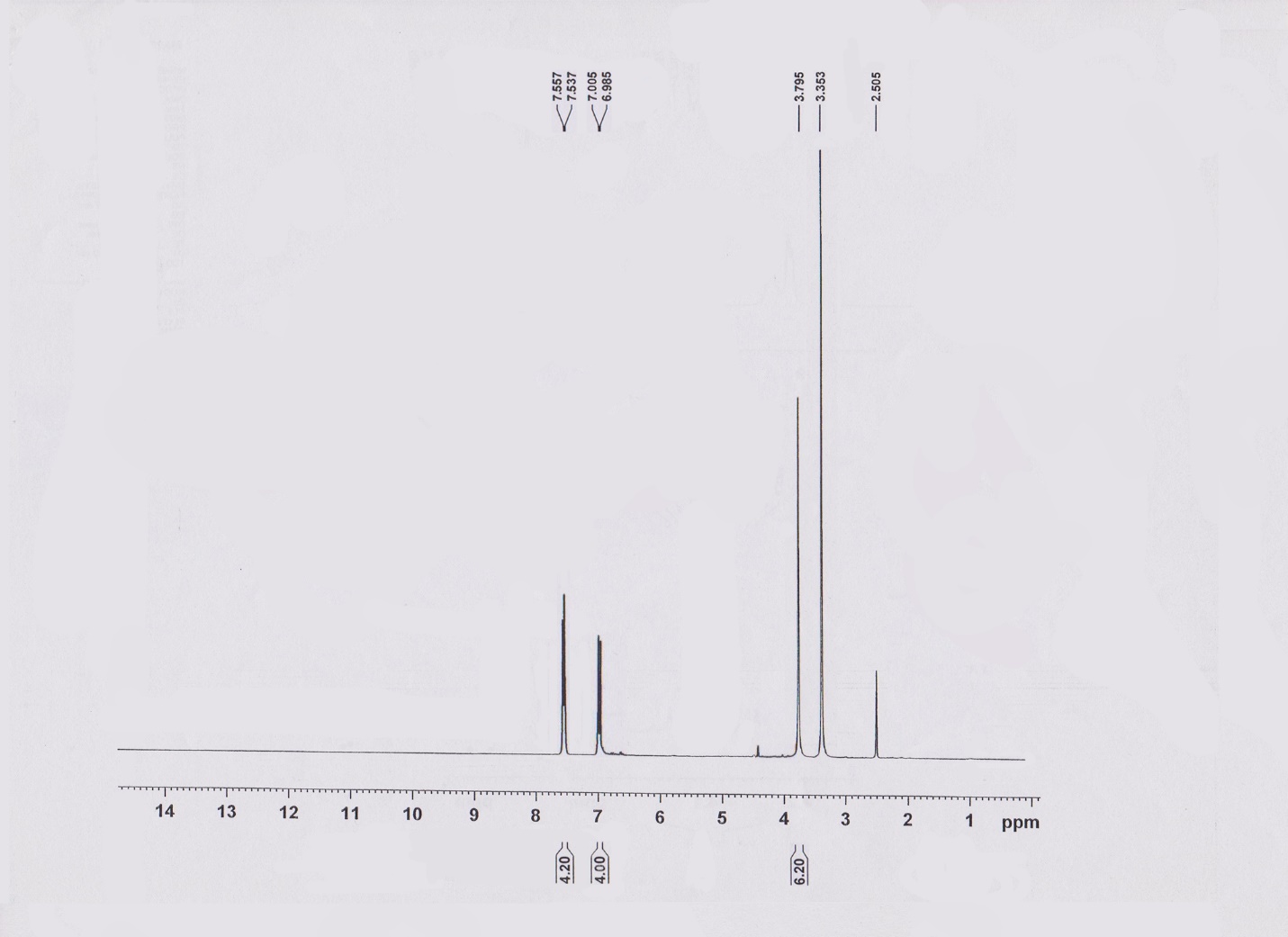


Figure 23. ^1^H NMR 4,4’-Dimethoxybiphenyl (6c)


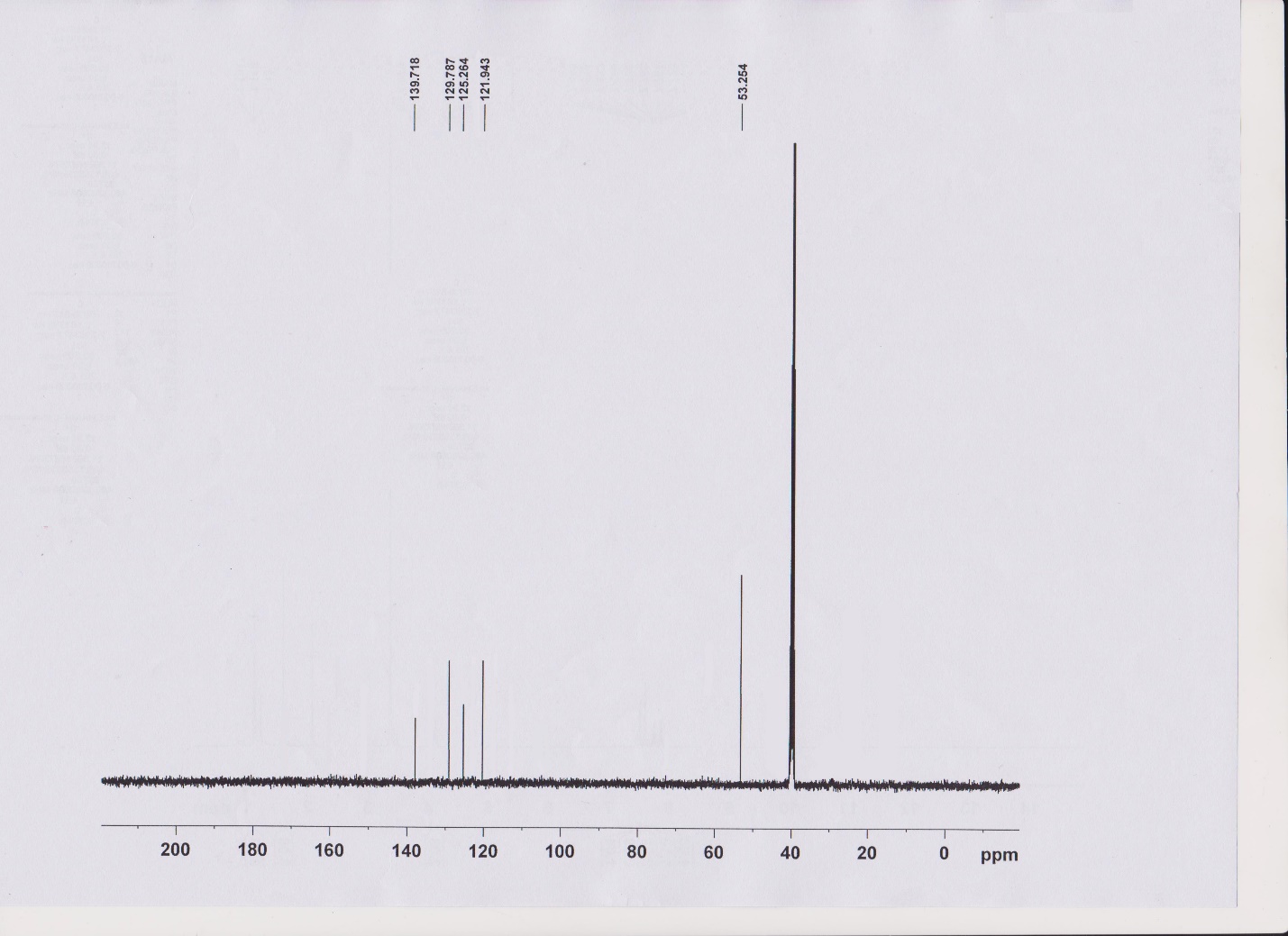


Figure 24. ^13^C NMR 4,4’-Dimethoxybiphenyl (6c)

2.8.3. 4,4’-Dimethoxybiphenyl (6c)

Pale yellow solid, m.p. 175–178 °C (Lit. [8]. 176–178 ºC); IR (KBr) Ѵ (cm^-1^): 3065 (C=C–H, stretch); 2957 (–C–H), 1521 (C=C_aromatic_), 1438 (C=C_aromatic_), 1248 and 1183 (C–O), 825 (C=C–H, bending); ^1^H NMR (DMSO-d_6_, 400 MHz) δ (ppm): 3.79 (s, 6H), 6.99 (d, J = 8.0 Hz, 4H, H_aromatic_), 7.54 (d, J = 8.0 Hz, 4H, H_aromatic_); ^13^C NMR (DMSO-d_6_, 100 MHz) δ (ppm): 53.2, 121.9, 125.2, 129.7, 139.7.


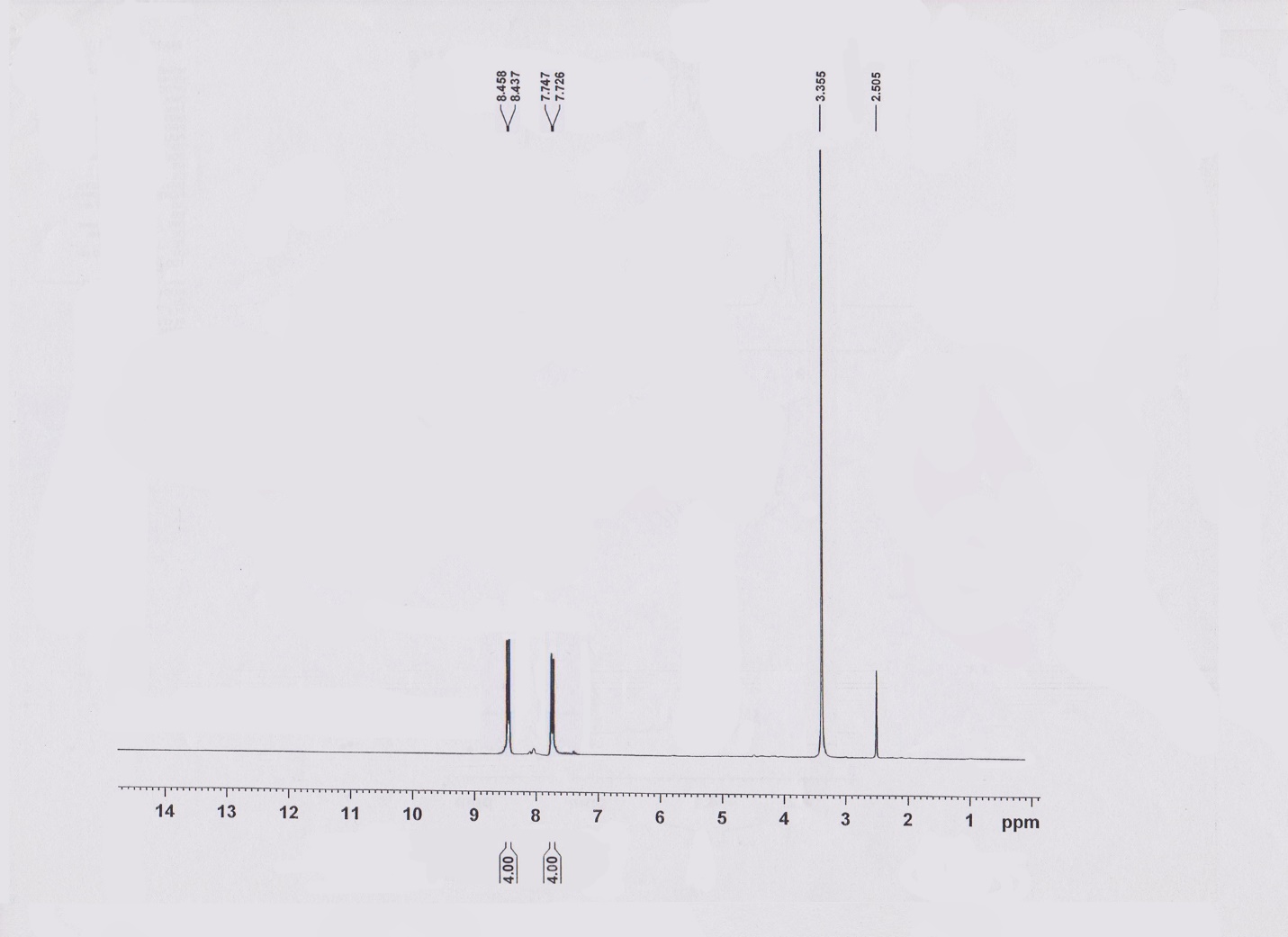


Figure 25. ^1^H NMR 4,4’-Dinitrobiphenyl (6d)


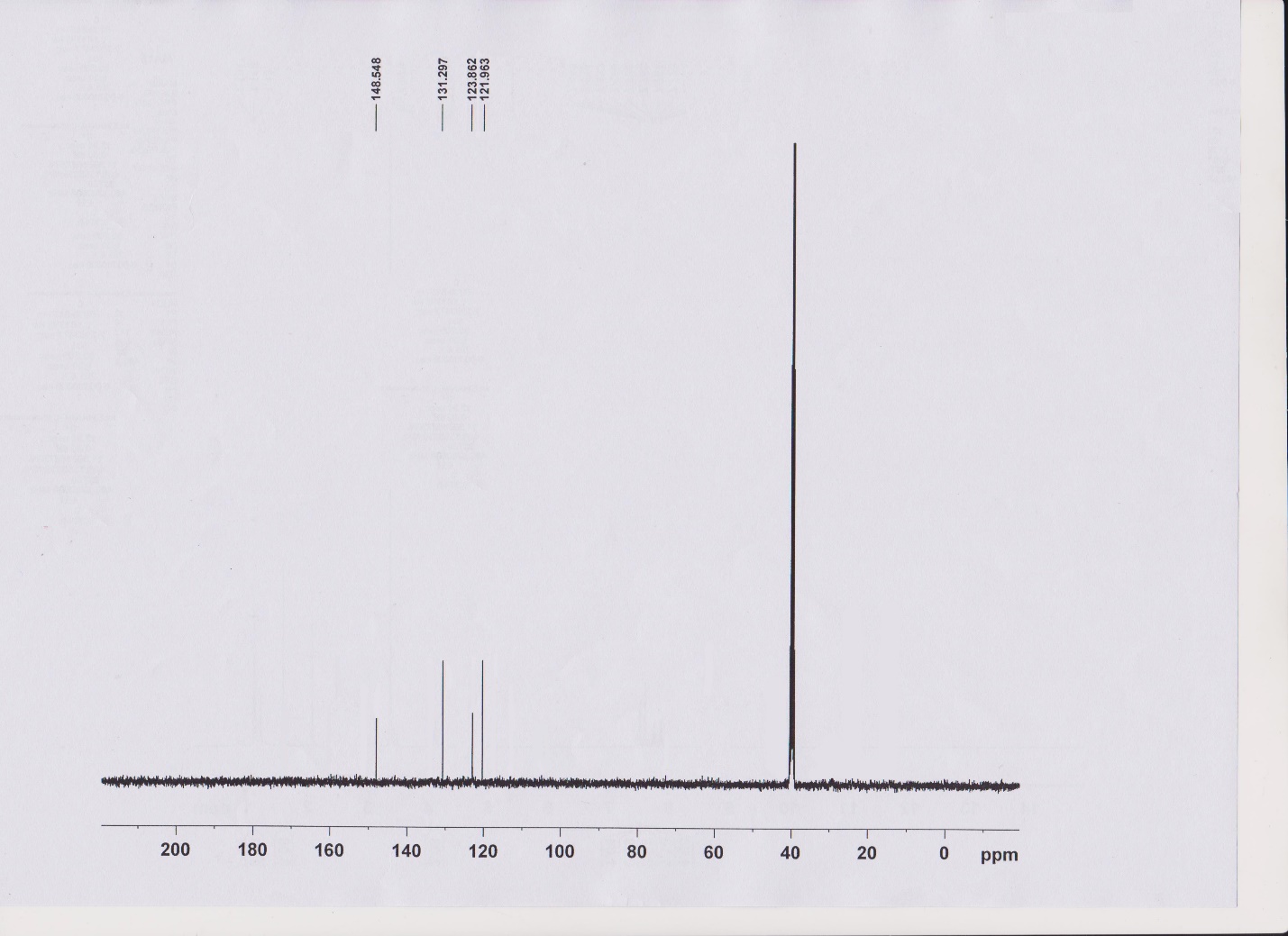


Figure 26. ^13^C NMR 4,4’-Dinitrobiphenyl (6d)

2.8.4. 4,4’-Dinitrobiphenyl (6d)

Yellow solid, m.p. 234–236 °C (Lit. [9]. 237–238 ºC); IR (KBr) Ѵ (cm^-1^): 3012 (C=C–H, stretch), 1599 (C=C_aromatic_), 1522 and 1351 (N–O, NO_2_), 847 and 693 (C=C–H, bending); ^1^H NMR (DMSO-d_6_, 400 MHz) δ (ppm): 7.73 (d, J = 8.4 Hz, 4H, H_aromatic_), 8.44 (d, J = 8.4 Hz, 4H, H_aromatic_); ^13^C NMR (DMSO-d_6_, 100 MHz) δ (ppm): 121.9, 123.8, 131.2, 148.5.


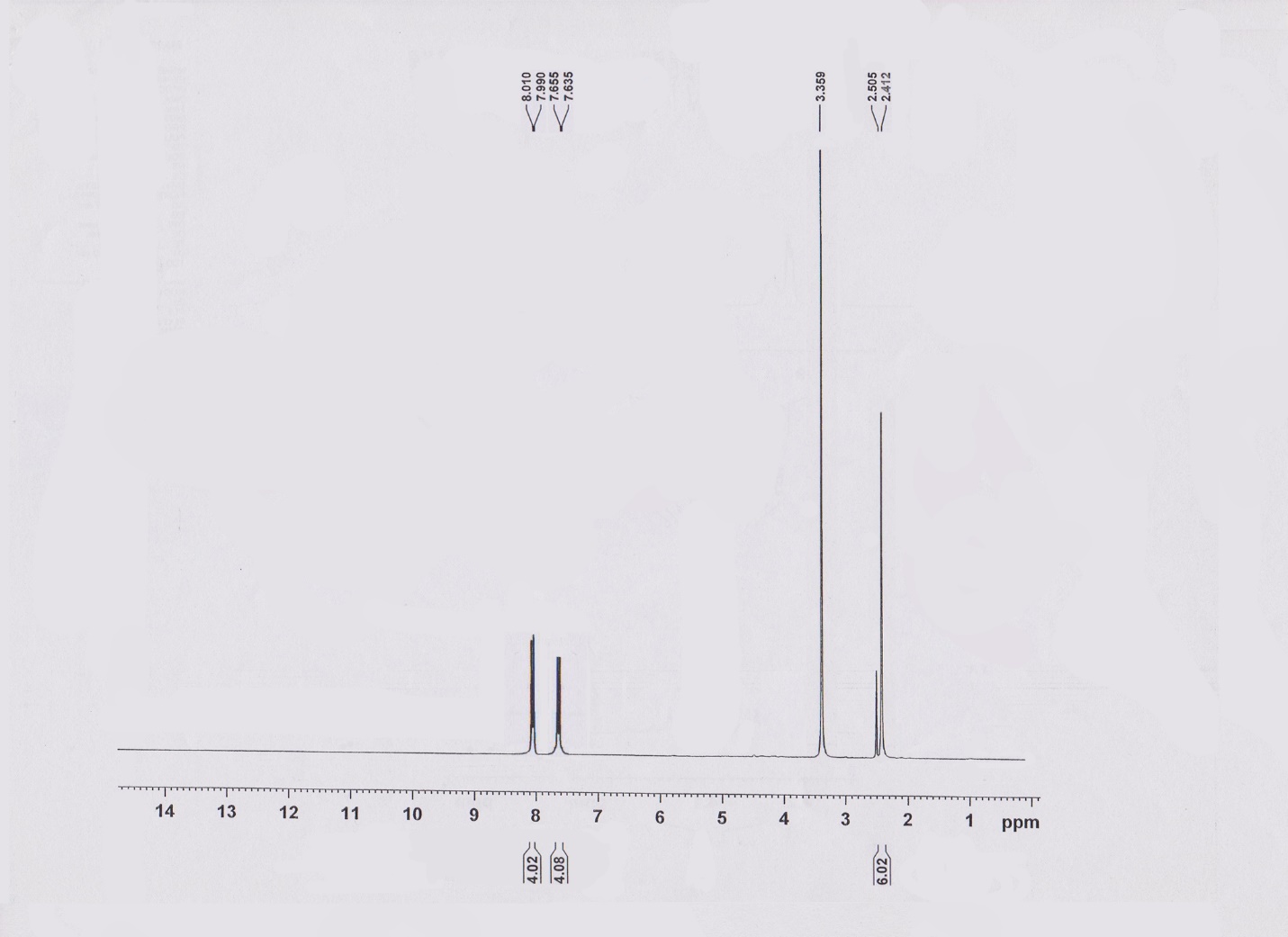


Figure 27. ^1^H NMR 4,4’-Diacetylbiphenyl (6e)


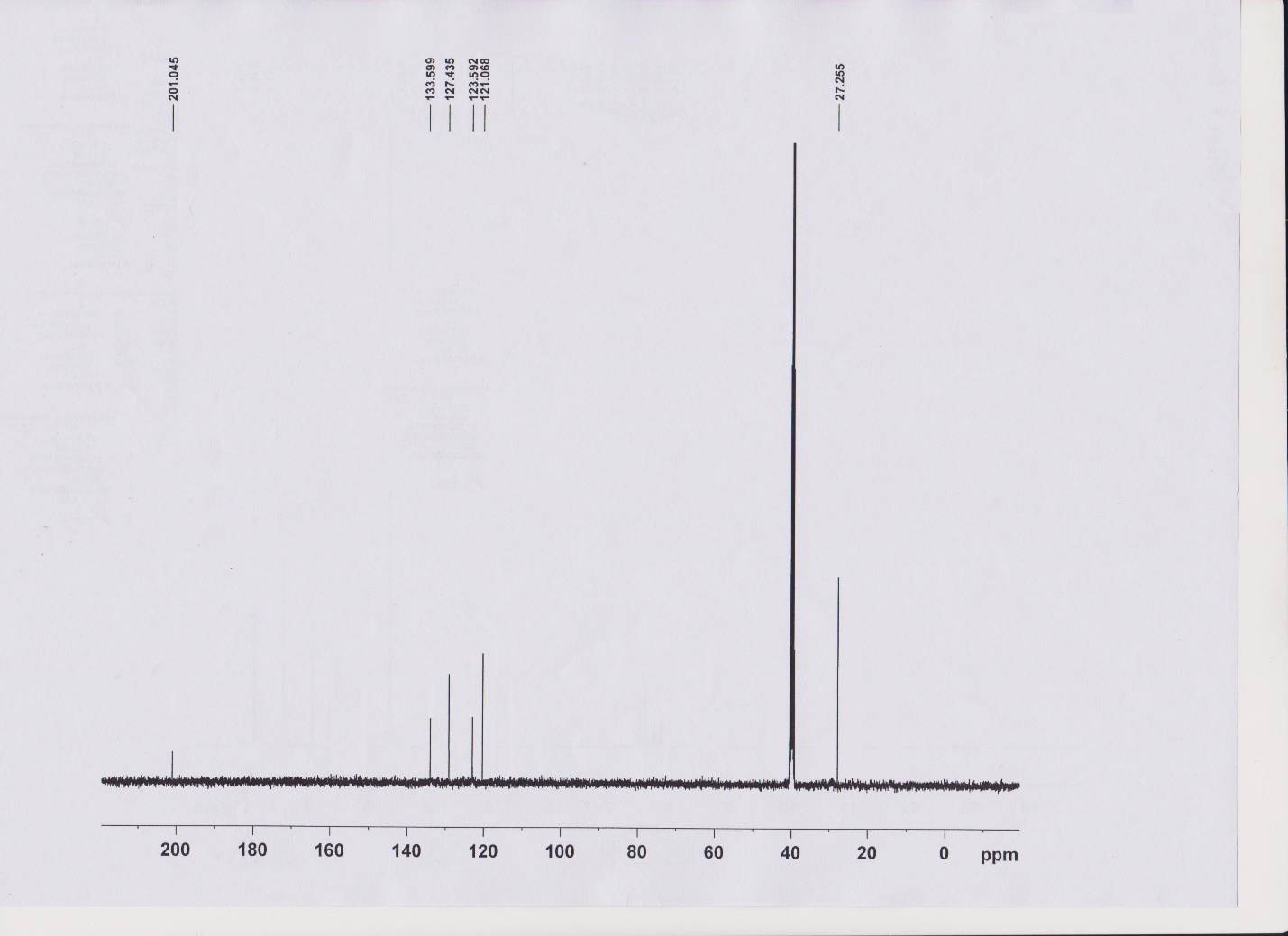


Figure 28. ^13^C NMR 4,4’-Diacetylbiphenyl (6e)

2.8.5. 4,4’-Diacetylbiphenyl (6e)

White solid, m.p. 193–194 °C (Lit. [10]. 190–192 ºC); IR (KBr) Ѵ (cm^-1^): 3054 (C=C–H, stretch), 2928 (–C–H), 1690 (C=O), 1615 (C=C_aromatic_), 809 (C=C–H, bending); ^1^H NMR (DMSO-d_6_, 400 MHz) δ (ppm): 2.41 (s, 6H), 7.64 (d, J = 8.0 Hz, 4H, H_aromatic_), 8.00 (d, J = 8.0 Hz, 4H, H_aromatic_); ^13^C NMR (DMSO-d_6_, 100 MHz) δ (ppm): 27.2, 121.0, 123.5, 127.4, 133.5, 201.0.

**Spectral data of catalyst:**

IR spectrum of catalyst after six times uses:


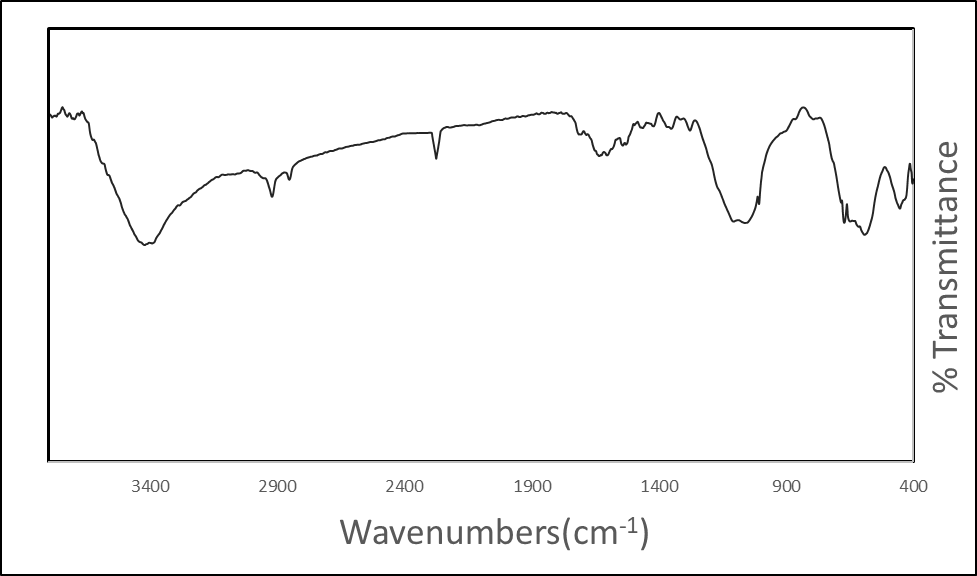


Figure 29. IR spectrum of catalyst after six times use

**References:**

1. Chassaing, S., et al., *“Click Chemistry” in Zeolites: Copper (I) Zeolites as New Heterogeneous and Ligand‐Free Catalysts for the Huisgen [3+ 2] Cycloaddition.* Chemistry–A European Journal, 2008. **14**(22): p. 6713-6721.

2. Abu‐Orabi, S.T., et al., *Dipolar cycloaddition reactions of organic azides with some acetylenic compounds.* Journal of heterocyclic chemistry, 1989. **26**(5): p. 1461-1468.

3. Ötvös, S.B., et al., *Alkyne–Azide Cycloadditions with Copper Powder in a High‐Pressure Continuous‐Flow Reactor: High‐Temperature Conditions versus the Role of Additives.* Chemistry–An Asian Journal, 2013. **8**(4): p. 800-808.

4. Naeimi, H., S. Dadashzadeh, and M. Moradian, *Facile and efficient sonochemical synthesis of 1, 4-disubstituted 1, 2, 3-triazole derivatives catalyzed by CuI under mild conditions.* Research on Chemical Intermediates, 2015. **41**: p. 2687-2695.

5. Aflak, N., et al., *Facile immobilization of copper (I) acetate on silica: A recyclable and reusable heterogeneous catalyst for azide–alkyne clickable cycloaddition reactions.* Polyhedron, 2019. **170**: p. 630-638.

6. Gonda, Z. and Z. Novák, *Highly active copper-catalysts for azide-alkyne cycloaddition.* Dalton Transactions, 2010. **39**(3): p. 726-729.

7. Matsuo, K., et al., *Nickel-Catalyzed Hydrodeoxygenation of Aryl Sulfamates with Alcohols as Mild Reducing Agents.* Synthesis, 2021. **53**(23): p. 4449-4460.

8. Yazdani, H. and A. Bazgir, *Lewis Acid Catalyzed Regio-and Diastereoselective Synthesis of Spiroisoxazolines via One-Pot Sequential Knoevenagel Condensation/1, 3-Dipolar Cycloaddition Reaction.* Synthesis, 2019. **51**(07): p. 1669-1679.

9. Zhou, Q., et al., *l-(−)-Quebrachitol as a ligand for selective copper (0)-catalyzed N-arylation of nitrogen-containing heterocycles.* The Journal of Organic Chemistry, 2019. **84**(12): p. 8160-8167.

10. Dabiri, M., et al., *Synthesis of gold nanoparticles decorated on sulfonated three‐dimensional graphene nanocomposite and application as a highly efficient and recyclable heterogeneous catalyst for Ullmann homocoupling of aryl iodides and reduction of p‐nitrophenol.* Applied Organometallic Chemistry, 2018. **32**(3): p. e4189.
